# Soil community history strengthens belowground multitrophic functioning across plant diversity levels in a grassland experiment

**DOI:** 10.1038/s41467-024-54401-z

**Published:** 2024-11-19

**Authors:** Angelos Amyntas, Nico Eisenhauer, Stefan Scheu, Bernhard Klarner, Krassimira Ilieva-Makulec, Anna-Maria Madaj, Benoit Gauzens, Jingyi Li, Anton M. Potapov, Benjamin Rosenbaum, Leonardo Bassi, Pamela Medina van Berkum, Ulrich Brose

**Affiliations:** 1 Institute of Biodiversity, Friedrich Schiller University Jena, Jena, Germany; 2 German Centre for Integrative Biodiversity Research (iDiv) Halle-Jena-Leipzig, Leipzig, Germany; 3 J.F. Blumenbach Institute of Zoology and Anthropology, University of Göttingen, Göttingen, Germany; 4 Institute of Biology, Leipzig University, Leipzig, Germany; 5 Centre of Biodiversity and Sustainable Land Use, University of Göttingen, Göttingen, Germany; 6 Institute of Biological Sciences, Cardinal St. Wyszynski University, Warsaw, Poland; 7 Senckenberg Museum für Naturkunde Görlitz, Görlitz, Germany; 8 Max Planck Institute for Chemical Ecology, Jena, Germany

**Keywords:** Community ecology, Plant ecology, Biodiversity, Ecosystem ecology

## Abstract

Biodiversity experiments revealed that plant diversity loss can decrease ecosystem functions across trophic levels. To address why such biodiversity-function relationships strengthen over time, we established experimental mesocosms replicating a gradient in plant species richness across treatments of shared versus non-shared history of (1) the plant community and (2) the soil fauna community. After 4 months, we assessed the multitrophic functioning of soil fauna via biomass stocks and energy fluxes across the food webs. We find that soil community history significantly enhanced belowground multitrophic function via changes in biomass stocks and community-average body masses across the food webs. However, variation in plant diversity and plant community history had unclear effects. Our findings underscore the importance of long-term community assembly processes for soil fauna-driven ecosystem function, with species richness and short-term plant adaptations playing a minimal role. Disturbances that disrupt soil community stability may hinder fauna-driven ecosystem functions, while recovery may require several years.

## Abstract (web summary)

Plant diversity and community history can jointly influence ecosystem functions, including those performed by soil fauna. This study shows that soil community history, rather than plant diversity or short-term plant adaptations, plays a crucial role in enhancing belowground ecosystem function.

## Introduction

The continuing anthropogenic disturbance of ecosystems is precipitating an alarming loss of species^1,2^, leading to local changes in species richness and community composition^3,4^. Experimental evidence from several studies has highlighted species diversity as an important driver of ecosystem functioning^5,6^, which is in turn tightly linked to services provided to humans^7^. Compared to monocultures, species-rich plant communities tend to exhibit higher functioning, such as increased primary productivity^8^, but also increased functioning of the consumer communities they support (e.g. decomposition^9,10^, herbivory and predation^9,11^). This positive biodiversity-ecosystem functioning (BEF) relationship has generally been attributed to complementarity effects^12–14^. That is, individuals in a species mixture have reduced niche overlap compared to individuals in monocultures, therefore the community makes more efficient use of the available resources^15,16^. Long-term experiments examining BEF relationships have found them to be weak or inconsistent in early years but strengthening over time to become more stable and stronger in more mature communities^17–20^. This indicates the essential roles of community assembly and co-adaptation of local populations in changes of ecosystem functioning as communities mature^15,21,22^.

During community establishment, ecosystem functioning can increase due to adaptations of the plant community to a specific abiotic and biotic environment. These adaptations can occur both within and between species^23,24^. Over successive generations, they lead to plant communities that consist of individuals whose traits allow them to coexist with other plants in the community, but also with the soil community it supports and interacts with^15^. If such adaptive changes increase productivity, the effect of these processes can then cascade to the soil community, resulting in a higher functioning soil food-web^25–27^.

Another important component contributing to ecosystem functions, such as nutrient cycling and population regulation^25,28^ is the soil fauna community that is supported by the plants. Soil fauna communities also undergo compositional changes over time during community assembly, in response to a specific biotic and abiotic environment^29^. These can involve replacement with potential increases or decreases in species diversity, changes in biomass in response to increased resource input by the plant community, or changes in the body-mass distribution. This, in turn, entails changes in the structure and function of the soil food web, which can lead to higher energy fluxes^30^, carbon and nutrient cycles^31^, and therefore increased overall ecosystem functioning^32^.

In contrast to well-documented temporal changes in the effects of plant diversity on plant-related functions, such as primary productivity or soil microbial activity, it is less well understood how the relationship between plant diversity and functions of the soil fauna community develops over time^15,21,22^. Soil fauna, with their diverse diets, span several trophic levels^25^. Their trophic activity includes herbivory, detritivory, microbivory and predation. Together, these trophic functions regulate processes such as nutrient release from detritus and plant growth^27,31^. The multitrophic functioning of the soil community is therefore an integral aspect of overall ecosystem functioning and essential in our understanding of how the latter develops over time^15,21^.

**Fig. 1:**
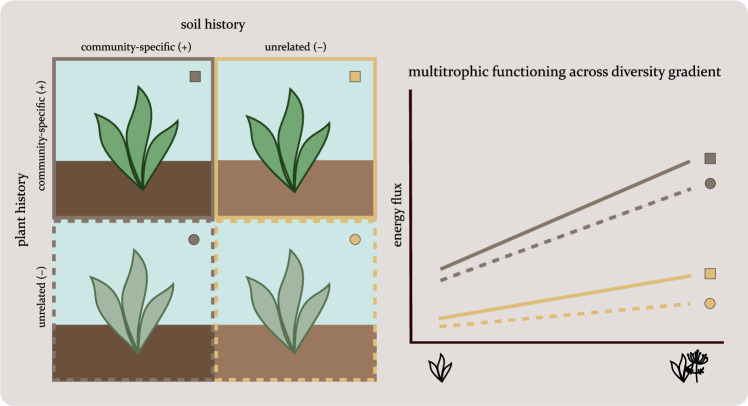
Experimental design and hypothesized relationships. Left: control mesocosm communities were composed of plants and soil derived from a reference field community with a decade of shared history. Treatment communities were lacking either community-specific plant history, soil history or both. Right: Soil fauna multitrophic functioning should be positively related to plant species diversity, but this relationship builds up over time. It would therefore be stronger in the control communities and would diminish when soil history or plant history are lacking.

Throughout the history of BEF studies, micro- and mesocosm experiments have been instrumental to develop a mechanistic understanding of the relationship between biodiversity and ecosystem processes^33–37^. They enable experimentation on properties that are difficult to manipulate in the field, such as plant community history and soil history independent of each other. We employed a large mesocosm experiment, combining plant diversity and community-specific plant and soil history, to understand their individual and interactive influence on ecosystem functioning performed by soil fauna (Fig. 1). Previous studies that examined the effects of plant diversity on the abundance or biomass of invertebrates^38,39^ have attempted to link changes of the invertebrate community to changes in its functions by assigning taxa to distinct trophic groups. However, the prevalence of omnivory, particularly among soil taxa, often impedes a clear trophic categorisation^40–42^. Using a food-web energetics approach, we can leverage the best information on soil fauna trophic preferences currently available^42^. This allows us to pivot from trophic groups to trophic links with a clear correspondence to functions such as herbivory or microbivory. The food web of a community as a whole integrates multiple trophic functions. Therefore, the total energy that flows through it can be used as a measure of multitrophic functioning of the community^43,44^. We expect that species-rich plant communities with shared plant and soil community-specific history will maintain high-functioning soil food webs; therefore, the experimental reduction of plant species or the removal of soil and plant community history will have detrimental effects on the multitrophic functions of the soil fauna community.

## Results

### Multitrophic soil fauna functioning depends on soil history rather than plant diversity or plant history

**Fig. 2:**
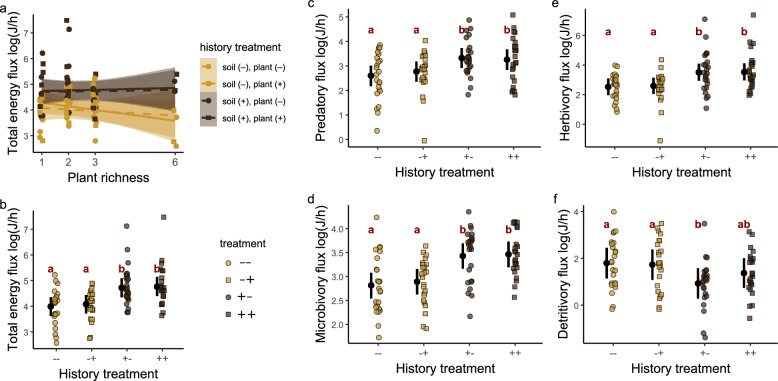
Energy flux of soil fauna food-webs. **a** The relationship of total energy flux with plant richness for different combinations of plant and soil history. **b** Total energy flux for the four community history manipulations. **c–f** Energy flux corresponding to predation, microbivory, herbivory and detritivory for the four community history manipulations. Black points are means with 95% HPD intervals. Groups with different red letters have mean differences whose uncertainty interval excludes zero. Across panels, *n* = 96.

Contrary to our hypothesis (Fig. 1), we did not find evidence of plant richness effects on the trophic functions of soil fauna communities, regardless of community history treatment (Fig. 2a). Therefore, we excluded effects of plant species richness from the subsequent analyses. Communities in mesocosms with plot-specific soil history had on average higher total energy flux compared to those with bare ground soil, regardless of the plant history treatment (difference with vs. without soil history: mean [95% HPD] = 0.71 [0.44, 0.98], Fig. 2b). This difference was largely reflected in fluxes related to individual trophic functions (predation: mean [95% HPD] = 0.6 [0.3, 0.93]; herbivory: mean [95% HPD] = 0.96 [0.53, 1.39]; microbivory: mean [95% HPD] = 0.59 [0.4, 0.785], Fig. 2c–e). The exception was detritivory, where mesocosms with soil but not plant history had on average lower detritivory fluxes than those with bare ground soil (mean [95% HPD] = −0.83 [−1.22, −0.455]) and marginally lower than mesocosms with soil and plant history (mean [90% HPD] = −0.44 [−0.87, −0.01]).

### Effects of soil history on belowground multitrophic functioning are mediated by changes in soil fauna body-mass and biomass

**Fig. 3:**
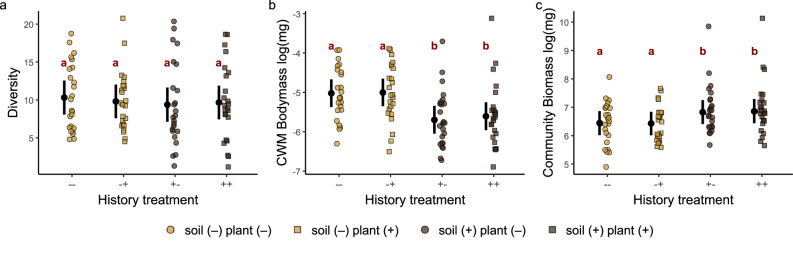
Soil fauna community properties. **a** Diversity (exponent of Shannon entropy), (**b**) community weighted mean body-mass and (**c**) soil fauna community biomass for the four community history manipulations. Black points are means with 95% HPD intervals. Groups with different red letters have mean differences whose uncertainty interval excludes zero. Across panels, *n* = 96.

We tested whether the differences in community energy fluxes that depend on the soil community history (Fig. 2) could find an explanation in shifts in diversity, the average body-masses or the cumulative biomass of the soil animals during community assembly. We found that there were no clear differences in soil fauna diversity between any combination of history treatments (Fig. 3a). However, soil fauna communities in mesocosms with plot-specific soil history exhibited on average lower community-weighted mean (CWM) body-mass than those without (difference with vs. without soil history: mean [95% HPD] = −0.64 [−0.94, −0.36], Fig. 3b), and higher biomass (difference with vs. without soil history: mean [95% HPD] = 0.41 [0.125, 0.705], Fig. 3c). The average differences in CWM body-mass were due to compositional shifts toward smaller taxa rather than reduction of body-mass within individual taxa (Supplementary Table 2). As expected, energy flux had a negative relationship with CWM body-mass (due to a higher metabolic rate of smaller organisms^45^; mean slope [95% HPD] = −0.29 [−0.35, −0.23], Supplementary Fig. 1a) and a positive one with biomass (mean slope [95% HPD] = 0.81 [0.74, 0.88], Supplementary Fig. 1b).

## Discussion

In a large Ecotron experiment, we found that under the same plant communities, soil fauna-driven ecosystem functioning is promoted if soil communities have a shared history with plant communities, in comparison to soil communities taken from bare ground. Surprisingly, our experiment did not confirm positive effects of either plant species richness or plant adaptation history on soil ecosystem functioning. Taken together, these results highlight the importance of animal community assembly and biomass accumulation for ecosystem functioning.

Our results do not support our hypothesis that plant communities of high species richness sustain higher functioning soil communities than communities with fewer plant species. This contrasts the findings of prior field experiments, a disparity that may be ascribed to various factors. Firstly, the relatively short duration of our experiment (i.e. 4 months) may be a contributing factor as it may limit the organic input and the accumulation of soil organic material in more diverse plant communities^46,47^, which is fueling soil food webs^48^. However, the lack of a detectable relationship in the shared soil and shared plant history treatment, where these processes should have already occurred in the field, reduces the plausibility of this explanation. Secondly, the disparity may be caused by the relatively short gradient in plant species richness (i.e. 1–6 plant species) compared to field experiments that cover a gradient in plant species richness up to 16^8^, 24^19^ or 60 species^49^. However, these relationships typically exhibit saturating increases, highlighting that the strongest effects of plant richness on ecosystem functioning occur at low diversity levels. Thirdly, unlike prior experiments, we kept equal plant density across all our mesocosms, whereas variation in plant density across diversity levels in field experiments can mediate plant diversity effects on productivity^50^. Fourthly, the disparity might be caused by the specific composition of the plant communities in our experiment. In this vein, simulation studies have shown that neutral to negative BEF relationships can arise from limited complementarity in plant resource use^16,51^, intraspecific competition exceeding interspecific competition^52^, or linkage patterns to higher trophic levels^51,53^. These findings also explain the substantial variation of BEF relationships in natural ecosystems that can also be neutral or even negative^54^, possibly explained by the presence of rare and non-native species^55^ or realistic diversity loss^56^. Overall, these arguments suggest differences in plant density and community composition between previous experiments and the present one as the most likely explanations for the lack of a positive relationship between plant species richness and soil ecosystem functioning in our study.

Additionally, we found that the average trophic functioning of soil fauna in communities with community-specific plant history was practically indistinguishable to those without plant history. In the context of this experiment, plant history refers to intergenerational adaptations of plant populations to a specific community of plants and soil biota through selection^23,24^. This in turn can lead to higher resource inputs (root tissue, exudates, litter) to the soil community. The absence of plant history effects on the soil fauna trophic functioning indicates that plant communities without such adaptations can support levels of soil fauna functioning comparable to adapted ones. Even if these adaptations lead to increased plant productivity, it may require time before this benefits the soil fauna community^29^. Conversely, the soil community can be shaped by its coexistence with a specific composition of plant species^57^. This includes soil microbes, whose composition is more strongly linked to plants than that of soil fauna. Thus, soil fauna could exhibit similar functioning when supported by plants of the same composition, even if the individual plants do not descend from the reference community.

Indeed, we found that fauna communities in soil with plant-community specific history had higher multitrophic functioning than those in soil from bare ground plots. The so-called bare ground plots in the Jena Experiment do not remain constantly bare; they are incidentally covered by plants that invade from the matrix species pool until periodically weeded out^49^. They therefore host opportunistic plant communities in constant reassembly. In contrast, the Trait-Based Experiment plots host plant communities that, despite invasions of non-target species (also continuously weeded out), have in the long run a fixed composition. The higher trophic fluxes in mesocosms with soil history are consistent with our hypothesis that ecosystem functioning increases over time. This increase can be related to soil organic matter build-up and soil fauna community assembly in response to a specific biotic and abiotic environment. The observed differences can therefore be attributed to legacy effects of plant inputs, combined with the more stable conditions under a fixed plant composition in the non-bare plots.

Generally, community assembly is a complex process including changes in species richness, shifts in community composition, and the built-up of biomass. Given the importance of soil community assembly for soil ecosystem functioning in our experiment, we have also addressed the relative importance of these three processes. In our experiment, the higher trophic functioning of fauna in soil with history was not related to more diverse soil communities. Instead, these communities were characterized by smaller average body-mass and higher total biomass. This decrease in average body size might be caused by more intensive colonization of soil pores during community assembly. This intensified colonization could be triggered by the establishment of more concentrated carbon pools, thereby increasing the availability of basal resources as well as soil porosity^58^, which in turn would also explain the higher biomasses in communities with soil history. Both of these shifts in community structure during assembly contribute to higher levels of ecosystem functioning. First, smaller-bodied organisms have higher mass-specific metabolism^45^. Second, higher biomass densities translate to higher energy flow necessary to maintain this biomass. Together, both processes first increase the population energy loss through metabolism and consequently also the energy fluxes through the feeding links that indicate ecosystem functions. Therefore, our results suggest that soil community assembly does not necessarily change species richness but may still change community composition with associated differences in body-mass and biomass that both result in increased community-level metabolic demands and consequently higher energy flow within the soil food-web.

The link between community assembly processes and community ecosystem functioning highlighted by our results also connects the local community functioning to meta-community processes at the landscape level^59^. The assembly of a soil community in a grassland patch depends not only on local processes but also on potential donor communities in the surrounding landscape. The assembly process can therefore be hindered by fragmentation or disturbance in surrounding patches^60^. Future studies should therefore shed light on how local ecosystem functioning changes over time, by examining the local influence of γ and β diversity of animal as well as plant communities under different scenarios of fragmentation or disturbance^61–63^. The relationship of local to patch-level diversity and composition implicates another aspect of plant community history, not considered here; namely, the order of arrival of different functional groups, such as grasses versus legumes or forbes, which can affect root deposition patterns^64^. These differences can in turn cascade to the functioning of the multitrophic soil community, either in terms of its vertical distribution in the soil or total levels of activity.

Overall, our study has revealed a clear effect of community specific soil history, through its influence on the soil animal community, on the ecosystem functions carried out by soil fauna food webs. As soil fauna diversity did not change significantly between the community history treatments, this is reinforcing the conclusion that community composition can be a stronger driver of ecosystem functioning than diversity^16,55,56^. Given that local diversity is not necessarily declining despite global biodiversity loss^3^, this finding suggests that ecosystem functioning may also be at risk if global change stressors lead to species redistribution and novel community compositions^65^. Our results highlight that such community reshuffling can disrupt pathways of community assembly with severe consequences for ecosystem functioning, independent of changes in diversity. They also underscore the importance of undisturbed soil for the functioning of grassland ecosystems

## Methods

### The JenaTron experiment

The experiment was conducted in the iDiv Ecotron platform, an indoor experimental mesocosm facility located in Bad Lauchstädt, Saxony-Anhalt, Germany, at the Experimental Research Station of the Helmholtz Center for Environmental Research (UFZ). It consists of 24 experimental units (EcoUnits) which can be partitioned into four isolated chambers. Each chamber can house a 50 cm Ø, 80 cm deep lysimeter in its lower “belowground” section and has an upper “aboveground” section that is 150 cm high. Inside the EcoUnits, abiotic conditions such as light and irrigation are controlled to simulate realistic conditions but with reduced environmental variability^66^. EcoUnits are spatially arranged in six experimental blocks.

In the spring of 2022, 48 soil monoliths were excavated from 23 plots of the Trait-Based Experiment (TBE), a long-term grassland BEF experiment (established in 2010^67^) that is part of the Jena Experiment^22^. The species composition of TBE plots is maintained by regular weeding three times a year. Two monoliths were excavated from each of 22 of the selected plots to cover a plant diversity gradient of 1, 2 and 3 species, and additionally four monoliths from a selected plot with 6 plant species. These monoliths were the basis of communities with community-specific soil history. Additionally, 48 soil monoliths were excavated from four bare ground plots of the Jena Experiment, which have been maintained without vegetation cover since 2002, also by weeding^49^. These monoliths formed the basis of communities without community-specific soil history. Both the TBE plots and bare ground plots were located at the same field and shared the same abiotic conditions. All monoliths were extracted from the field using the steel lysimeters as corers, equipped with a rotating cutting system on the bottom edge. Existing vegetation, as well as 5 cm of topsoil containing the seedbank, were removed before establishing the mesocosm communities. Each EcoUnit housed four monoliths; two with soil communities sharing history with a reference plant community and two without a shared history with the plant community (i.e. bare ground monoliths). The soil history treatment was then crossed with a plant history treatment. Two of the monoliths were planted with pre-grown seedlings coming from the reference community (seeds were collected in 2019, i.e. 9 years after the experiment was established, representing a plant history treatment). The other two were planted with seedlings of the same species but from the seed material used in the establishment of the TBE (representing no plant history treatment). Seedlings were pre-grown in a greenhouse, then transplanted to the monoliths. Therefore, each EcoUnit had one mesocosm with community-specific soil and plant history, one with soil but not plant history, one with plant but not soil history and one with neither soil or plant history, while all four had the same plant species composition at equal plant community density. The experiment ran from June to October 2022. Any emerging seedlings were weeded out over the first couple of weeks so that only transplanted target plants with the respective plant history were kept.

### Soil fauna sampling and measurements

At the end of the experiment, we extracted soil cores to assess the density of soil fauna in each mesocosm. We used one 15 cm Ø core for macrofauna, one 5 cm Ø core for mesofauna and three pooled 2 cm Ø cores for microfauna (nematodes). All cores were taken to 10 cm depth. We used heat extraction to extract mesofauna and macrofauna^68,69^ from the respective cores, and stored animals in 65% ethanol. Nematodes were extracted from 20 g of fresh soil using a modified Baermann-funnel method^70^ and stored in 4% formalin.

The extracted nematodes were counted and up to 100 individuals were identified at genus level. The abundance of nematodes in each mesocosm was calculated based on the grams of dry soil per 20 grams of fresh soil and dry soil density in each mesocosm. We used the taxonomic composition of the identified individuals to calculate the abundance of the different taxa in each mesocosm. We retrieved feeding preferences and body-mass information of the different taxa from Nemaplex^71^. Mesofauna and macrofauna were identified at family level. We measured the body length (and width for macrofauna) of up to 10 individuals per taxon per mesocosm and used group-specific length-mass^72^ and length-width-mass^73^ regressions to calculate body-mass. The abundance of the different taxa in each mesocosm was calculated based on the surface of the soil core and the surface of the mesocosm.

### Calculation of energy flux

To assess the trophic activity of soil fauna, we calculated the flux of energy across the feeding links of the food webs composed by the soil fauna communities of the 96 mesocosms using the fluxweb R package^74^. The rationale of this method is detailed in refs. ^43,45^. Under a steady state assumption, energy lost from each node in the food web due to metabolism or consumption by its consumers is compensated by energy gained from the node’s resources. Assimilation efficiencies are used to take account of the fact that not all biomass consumed from a resource can be metabolized by its consumer^75^. So, the building blocks required to calculate fluxes are population metabolic losses, an interaction matrix and assimilation efficiencies.

We calculated metabolic losses for an average individual of each population as a function of body-mass (after^76^). For this, we employed the average body-mass of each taxon in each mesocosm. We then used densities to extrapolate to population level losses (i.e. population level energy loss to metabolism equals the average individual metabolic rate times the number of individuals in the population).

We constructed mesocosm-specific trophic interaction matrices based on the following procedure. General consumer preferences for animal, plant, microbial, or detrital diet were based on^77^ for oribatid mites and on^42^ for other soil fauna. Each consumer could feed on multiple food resources with proportions from 0 to 1. For predatory interactions, a consumer’s expected animal diet composition was then refined as proposed in ref. ^44^, by considering predator-prey mass ratios, prey agility and possession of physical or chemical defenses, vertical stratification in the soil and finally the relative biomass of different prey taxa. We used the model proposed by ref. ^78^, (with re-estimated coefficients restricted to terrestrial invertebrates’ interactions in the GATEWAy dataset; see Supplementary Note 1) to calculate the probability that a predator of a certain size will consume prey taxa of different sizes. In the resulting matrices, the elements m_ij_ of each matrix have positive values if consumer *j* feeds on resource *i* and zero otherwise. The normalized elements of each vector *j* sum to one, expressing each consumer’s expected diet. Finally, we assumed an assimilation efficiency of 90.6% for predation and microbivory, 54.5% for herbivory and 15.8% for detritivory^75^.

We calculated the total energy flux of the soil fauna community as the sum of energy that flows across all the links of a mesocosm’s food-web. This provides a proxy of the multitrophic functioning of the community. We also summed energy flows from individual resource types, representing functions of the community, i.e. herbivory, detritivory, microbivory and predation. To understand what is driving differences of energy flux among treatments, we also considered the biomass of each soil fauna community, its community weighted mean (CWM) of body-mass, and its diversity H’, quantified as the exponent of Shannon entropy, based on the relative biomass of the different taxa.

### Statistical analysis

We started our analysis with a general model for each of the aggregate fluxes and community metrics as a response variable and plant species richness (standardized to zero mean and unit variance), community history (a four-level categorical variable) and their interaction as fixed effects, as well as block and EcoUnit (nested in block) as random effects. We subsequently simplified our models, retaining only community history and random effects. Where appropriate, we used a Student-t distribution instead of Gaussian, to minimize the influence of outliers^79^. Models were fitted in Stan via the brms package^80^, using default priors and four MCMC chains with 8,000 iterations each (the first half used as warm-up). We validated model convergence with visual inspection of chain mixing as well as R-hat values (≤1.01) and model fit with posterior predictive checks. Responses were log-transformed when posterior predictive checks indicated skewed distributions that were not fitted well by the model. We examined pairwise contrasts of the four community history treatments using the emmeans package^81^. We use the exclusion of zero from the 95% and 90% highest posterior density intervals (HPD) to assess how statistically clear the estimated differences are^82^.

### Sensitivity analysis

We conducted two sets of sensitivity analyses to evaluate the robustness of our findings. In the first one (Supplementary Table 2), we recalculated energy fluxes, fauna community biomass and CWM body-mass while ignoring body-mass differences of taxa across mesocosms. In other words, for each taxon, we used the average across mesocosms body-mass, instead of mesocosm-specific body-mass. We did so to investigate to what extent the observed differences between treatments can be attributed to within taxon shifts in body-mass or community-level changes that are based on changes of abundance of different taxa. In the second one (Supplementary Note 2 Supplementary Fig. 2), we examined the influence of variation of population biomass and, to relax the steady state assumption underlying our flux calculation, of metabolism. Our main findings were robust to these perturbations.

### Reporting summary

Further information on research design is available in the Nature Portfolio Reporting Summary linked to this article.

## Supplementary information

Supplementary Information

Reporting Summary

Transparent Peer Review file

## Data Availability

Data are available in the GitHub repository https://github.com/amynang/jenatron_soilfoodwebs and archived in Zenodo (10.5281/zenodo.13923794). All figures can be reproduced using the script “analysis_main.R” in the deposited code.

## Code availability

Code is available in the GitHub repository https://github.com/amynang/jenatron_soilfoodwebs and archived in Zenodo (10.5281/zenodo.13923794).
