# Supplementary material for: Soil community history strengthens belowground multitrophic functioning across plant diversity levels in a grassland experiment
Source: Nat Commun. 2024 Nov 19;15:10029. doi: 10.1038/s41467-024-54401-z (PMC11577027; doi:10.1038/s41467-024-54401-z)
Supplement: Supplementary file 1 — Supplementary Information [file 41467_2024_54401_MOESM1_ESM.pdf]

Soil community history strengthens belowground multitrophic functioning across plant diversity levels in grassland experiment

SUPPLEMENTARY INFORMATION

Supplementary Note 1

Probability of prey consumption based on predator-prey mass ratio

We have used the model proposed by (1):

$$P_{ij} = \theta_i exp\left(-\frac{(m_i - m_j - \mu_i)^2}{2\sigma_i^2}\right) \quad (1)$$

$$\mu_i(m_i) = \beta_{\mu 0} + \beta_{\mu 1} m_i \quad (2)$$

$$\sigma_i^2(m_i) = exp\left(\beta_{\sigma^2 0} + \beta_{\sigma^2 1} m_i\right) \quad (3)$$

$$\theta_i(m_i) = \frac{exp(\beta_{\theta 0} + \beta_{\theta 1} m_i)}{1 + exp(\beta_{\theta 0} + \beta_{\theta 1} m_i)} \quad (4)$$

Where P_{ij} is the probability that predator *i* consumes prey *j*, m_i and m_j are their log_{10}(g) body-masses, μ_i is the optimal log_{10} size ratio for predator *i*, θ_i is the feeding probability when prey *j* matches the optimal size ratio. The coefficients (Table S1) were re-estimated after restricting the data used in (1) to invertebrate-invertebrate interactions in terrestrial food webs.

Table S1. The coefficients of the model

	mean	2.50% CrI	97.50% CrI
$\beta_{\mu 0}$	0.35	0.3453	0.3548
$\beta_{\mu 1}$	-0.2235	-0.2278	-0.2192
$\beta_{\sigma^2 0}$	-0.1167	-0.1225	-0.111
$\beta_{\sigma^2 1}$	-0.0106	-0.0161	-0.0049
$\beta_{\theta 0}$	0.3742	0.3698	0.3784
$\beta_{\theta 1}$	0.0105	0.060	0.0150

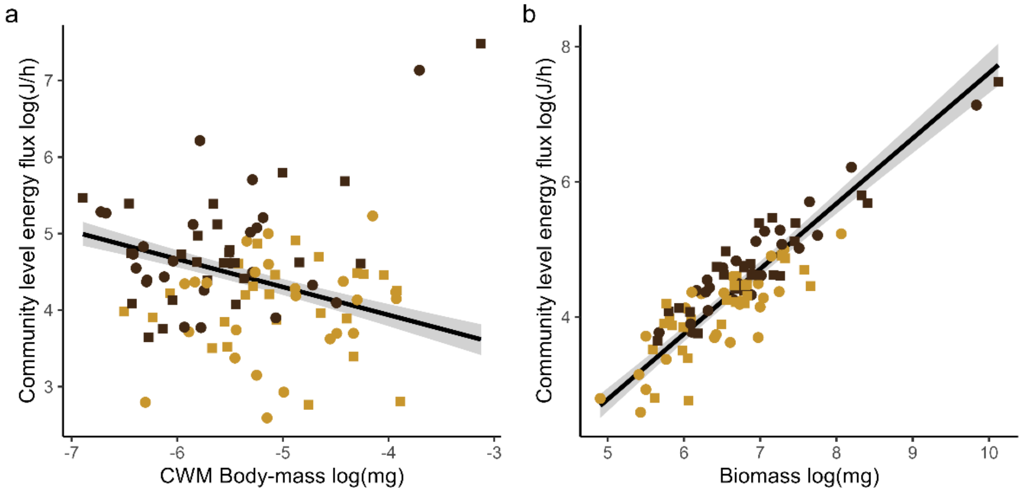

Figure S1 Marginal effects of community weighted mean body-mass and biomass on community level energy flux. These relationships explain why the observed differences in average body-mass and fauna community body-mass shown in Fig. 3 of the main text give rise to the difference in energy flux shown in Fig1b. Color differentiates soil history treatments: community specific soil (brown), unrelated soil (yellow). Shape differentiates plant history treatments: community specific plant history (square), unrelated (circle). Solid lines are mean estimates for the average relationship bound by 95% uncertainty intervals. In both panels n=96.

Table S2 Sensitivity of main findings to the method of body-mass estimation. For our main analysis, fluxes, fauna community biomasses and cwm body-masses were calculated based on mesocosm specific macro and mesofauna taxa body-mass information. Here we contrast the results of that analysis to ones produced based on the average across mesocosms body-mass of each taxon, i.e. ignoring body - mass differences across mesocosms. The top half of the table repeats the estimates and credible intervals reported in the main text. The bottom half shows the respective estimates and intervals for the reanalysis.

**main analysis – mesocosm specific body-mass**				
response	*contrast*	*mean*	*lower*	*upper*
total flux	with – without soil history	0.71	0.44	0.98
predation	with – without soil history	0.6	0.3	0.93
herbivory	with – without soil history	0.96	0.53	1.39
microbivory	with – without soil history	0.59	0.4	0.79
detritivory	with soil, no plant history – without soil history	-0.83	-1.22	-0.46
detritivory	with soil, no plant history – with soil and plant history	-0.44	-0.87	-0.01
fauna biomass	with – without soil history	0.41	0.13	0.71
cwm body-mass	with – without soil history	-0.64	-0.94	-0.36
**reanalysis – across mesocosms body-mass**				
response	*contrast*	*mean*	*lower*	*upper*
total flux	with – without soil history	0.7	0.44	0.96
predation	with – without soil history	0.54	0.24	0.86
herbivory	with – without soil history	0.95	0.54	1.39
microbivory	with – without soil history	0.58	0.40	0.78
detritivory	with soil, no plant history – without soil history	-0.88	-1.31	-0.44
detritivory	with soil, no plant history – with soil and plant history	-0.51	-0.94	-0.09
fauna biomass	with – without soil history	0.40	0.13	0.68
cwm body-mass	with – without soil history	-0.65	-0.94	-0.36

Supplementary Note 2

Analysis of sensitivity of main findings to variation of community properties

We examined the sensitivity of our main findings to variations of biomass and metabolic losses. To do so, we perturbed two fundamental properties of the observed communities. First, we randomly sampled abundance of the taxa in each community from the interval [$abundance_{obs}$ – 0.2* $abundance_{obs}$, $abundance_{obs}$ + 0.2*$abundance_{obs}$]. Perturbations of abundance result in perturbations of biomass (biomass = abundance*body-mass), which itself feeds into the interaction matrix (as predatory interactions depend on the relative biomass of prey taxa). They also result in perturbations of population-level metabolic losses ($Loss_{pop}$ = $Loss_{ind}$*abundance). We additionally perturbed individual metabolic losses by drawing from the interval [$Loss_{ind}$ – 0.2* $Loss_{ind}$, $Loss_{ind}$ + 0.2* $Loss_{ind}$]. This variation in the population-level loss term permits to relax the equilibrium assumption, by adding a new component to the loss term that is equivalent to a change in biomass per unit of time. For consumer *i,* the energy gains (G_i) through consumption and assimilation efficiency, are balanced by losses through predation (P_i) and metabolism (L_i) as well as an extra term representing an additional loss or gain of energy (ΔL_i) which can represent changes in species (because of the direct relationship between joules and biomass):

$$G_i = P_i + L_i + \Delta L_i. \qquad (5)$$

Using this approach, we generated 250 versions of our communities or in other words, 250 versions of the experimental data. We then repeated our analysis for each of the 250 versions. Here we report on the variation of mean estimates and credible intervals of the results reported in the main text.

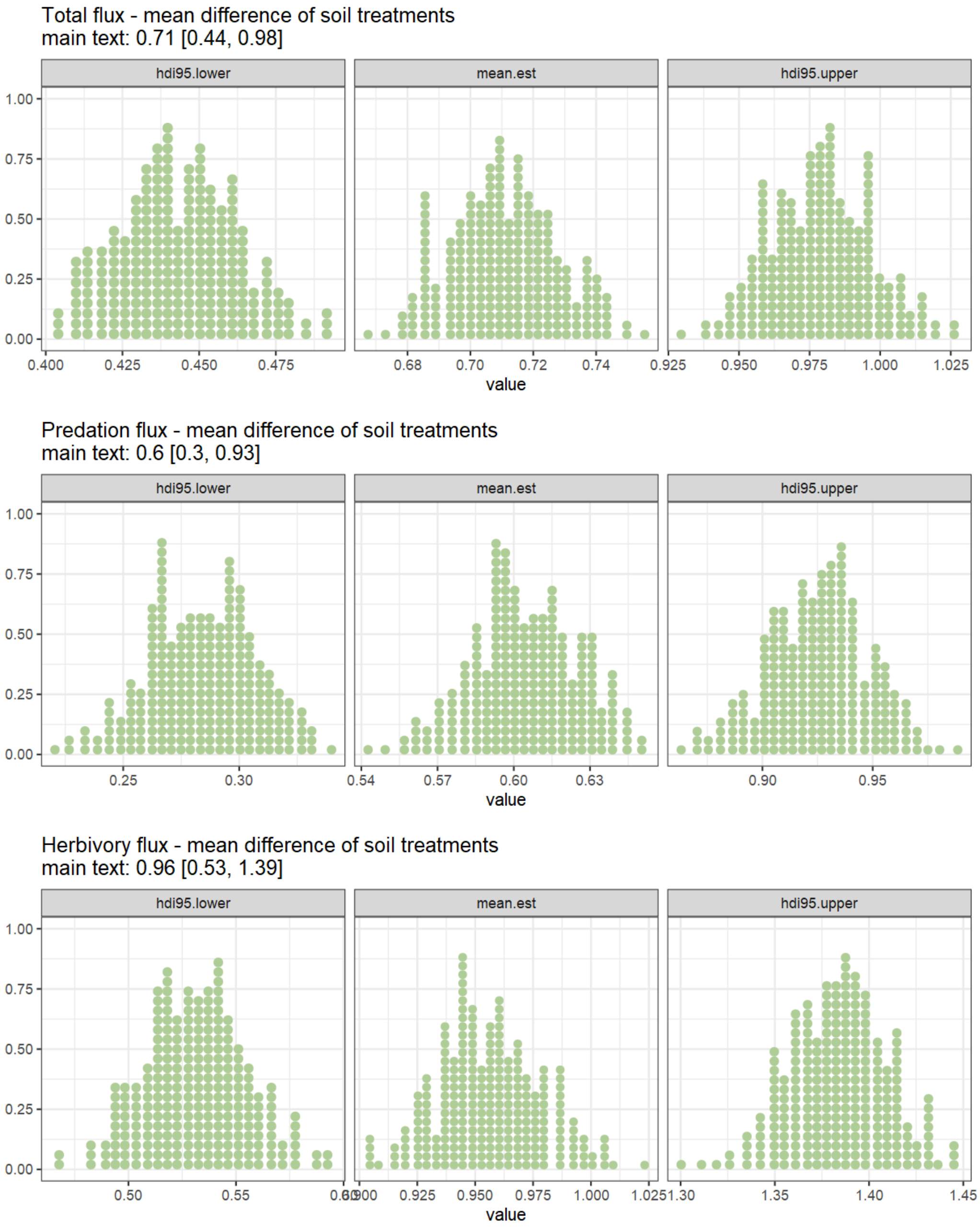

Figure S2 Variation of the mean estimate and lower and upper bounds of the credible interval of differences between history treatments, across the 250 versions (each dot corresponds to one version). Above each set of figures is shown the estimate reported in the main text. Red color highlights those instances where the credible interval contains zero.

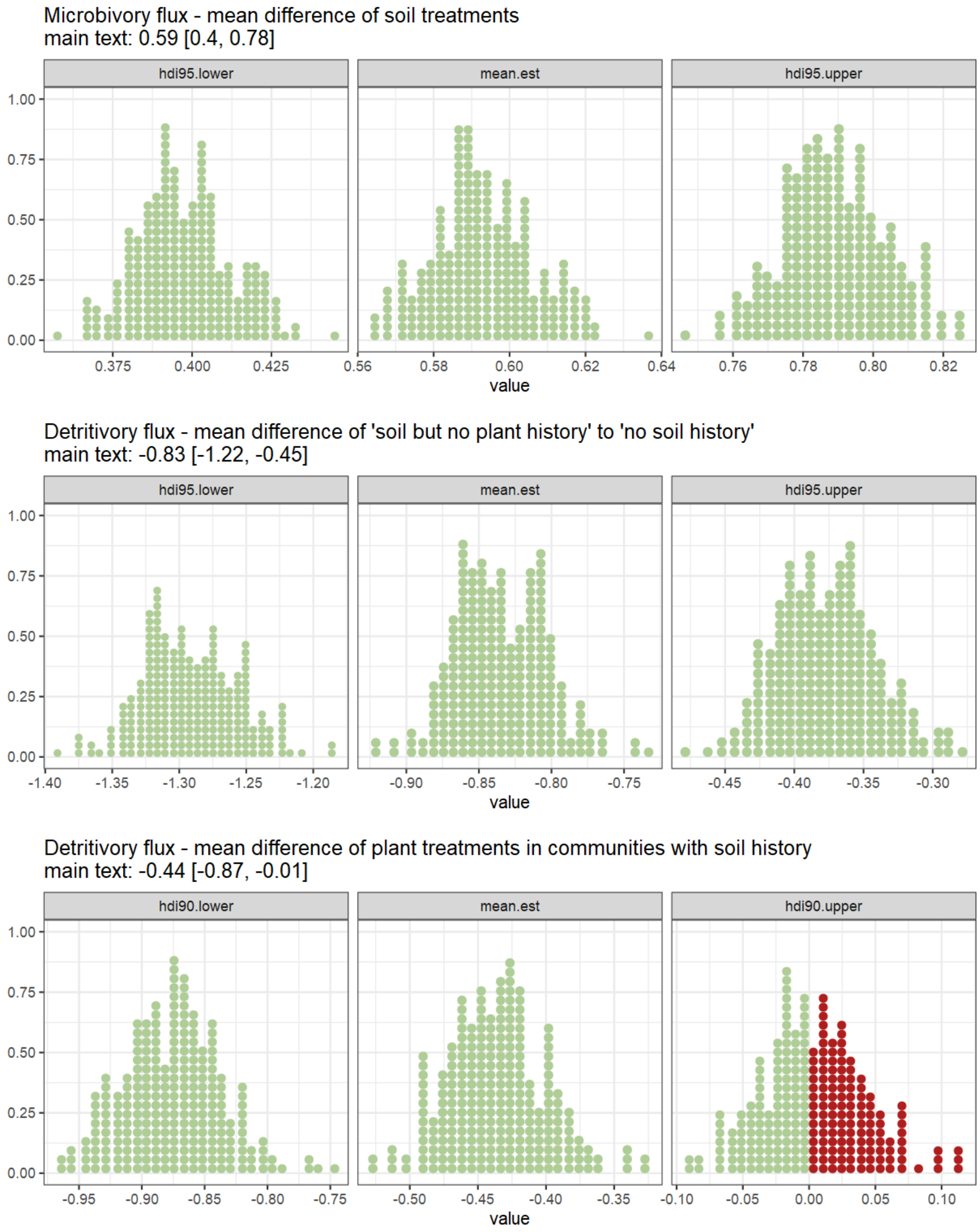

FigureS2 cont.

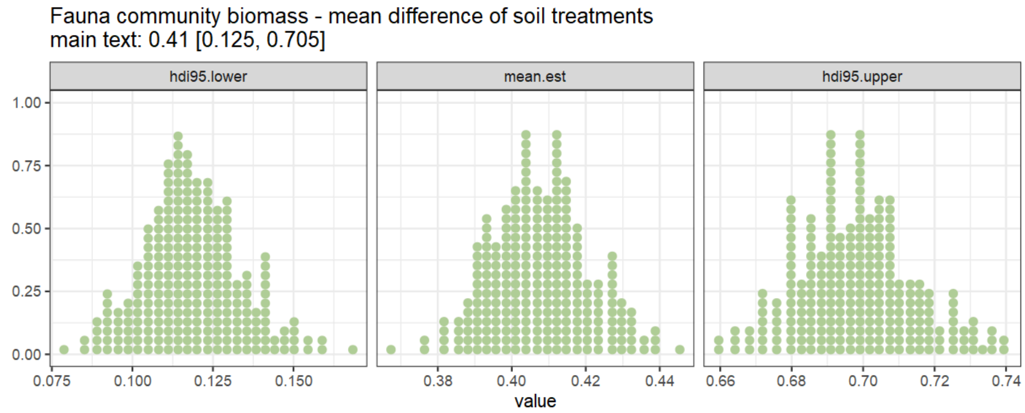

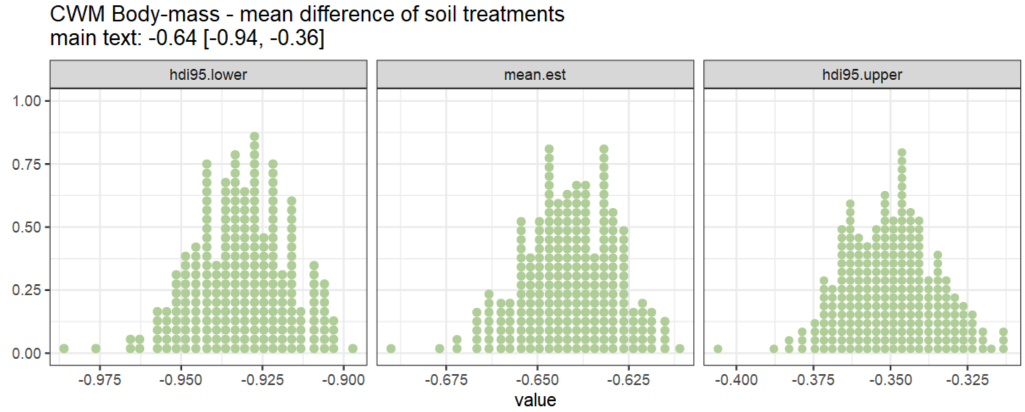

Figure S2 (cont.)

Table S3 Average abundance of broad groups across the experimental mesocosms. Taxa have been aggregated to broad groups. The detailed list of taxa abundance in each mesocosm is available in the supplementary data files.

Taxonomic group	Average abundance
Herbivorous nematodes	122321.68
Microbivorous nematodes	61888.23
Omnivorous nematodes	31522.56
Predatory nematodes	14108.97
Collembola	6156.25
Oribatid mites	4657.22
Prostigmatid mites	1354.07
Mesostigmatid mites	846.24
Astigmatid mites	23.73
Protura	167.71
Symphyla	78.13
Pauropoda	66.67
Diplura	6.37
Chilopoda	79.75
Hemiptera	73.61
Diplopoda	56.25
Staphylinidae	8.80
Other coleoptera	6.25
Diptera	4.40
Gastropoda	3.82
Spiders	1.74

Table S4 The invertebrate taxa that comprise the soil food webs of the JenaTron mesocosms. Agility, physical protection, metabolites and the vertical stratification of each taxon is from (2). The first three attributes are used as weights, so that when <1, they decrease the probability of consumption of that taxon as prey. Vertical stratification is used to construct a Bray-Curtis dissimilarity matrix, which again modifies consumption probability based on the overlap of predator and prey.

taxon_name	group	class	order	family	Agility	Physical	Metabolites	above	epi	hemi	eu
Amphidelus	Nematodes	Enoplea	Enoplida	Alaimidae	1	1	1	0	0	1	1
Cervidellus	Nematodes	Chromadorea	Rhabditida	Cephalobidae	1	1	1	0	0	1	1
Eumonhystera	Nematodes	Chromadorea	Monhysterida	Monhysteridae	1	1	1	0	0	1	1
Prismatolaimus	Nematodes	Enoplea	Triplonchida	Prismatolaimidae	1	1	1	0	0	1	1
Bastiania	Nematodes	Enoplea	Triplonchida	Bastianiidae	1	1	1	0	0	1	1
Rhabdolaimus	Nematodes	Enoplea	Enoplida	Rhabdolaimidae	1	1	1	0	0	1	1
Acrobeloides	Nematodes	Chromadorea	Rhabditida	Cephalobidae	1	1	1	0	0	1	1
Paramphidelus	Nematodes	Enoplea	Enoplida	Alaimidae	1	1	1	0	0	1	1
Acrolobus	Nematodes	Chromadorea	Rhabditida	Cephalobidae	1	1	1	0	0	1	1
Eucephalobus	Nematodes	Chromadorea	Rhabditida	Cephalobidae	1	1	1	0	0	1	1
Panagrolaimus	Nematodes	Chromadorea	Rhabditida	Panagrolaimidae	1	1	1	0	0	1	1
Prodesmodora	Nematodes	Chromadorea	Microlaimida	Microlaimidae	1	1	1	0	0	1	1
Alaimus	Nematodes	Enoplea	Enoplida	Alaimidae	1	1	1	0	0	1	1
Tylocephalus	Nematodes	Chromadorea	Plectida	Plectidae	1	1	1	0	0	1	1
Chiloplacus	Nematodes	Chromadorea	Rhabditida	Cephalobidae	1	1	1	0	0	1	1
Acrobeles	Nematodes	Chromadorea	Rhabditida	Cephalobidae	1	1	1	0	0	1	1
Cephalobus	Nematodes	Chromadorea	Rhabditida	Cephalobidae	1	1	1	0	0	1	1
Protorhabditis	Nematodes	Chromadorea	Rhabditida	Rhabditidae	1	1	1	0	0	1	1
Desmoscolex	Nematodes	Chromadorea	Desmoscolecida	Desmoscolecidae	1	1	1	0	0	1	1
Aulolaimus	Nematodes	Chromadorea	Plectida	Aulolaimidae	1	1	1	0	0	1	1
Plectus	Nematodes	Chromadorea	Plectida	Plectidae	1	1	1	0	0	1	1
Heterocephalobus	Nematodes	Chromadorea	Rhabditida	Cephalobidae	1	1	1	0	0	1	1
Microlaimus	Nematodes	Chromadorea	Microlaimida	Microlaimidae	1	1	1	0	0	1	1

Anaplectus	Nematodes	Chromad orea	Plectida	Plectidae	1	1	1	0	0	1	1
Cylindrolaimus	Nematodes	Chromad orea	Araeolaimida	Diplopeltidae	1	1	1	0	0	1	1
Mesorhabditis	Nematodes	Chromad orea	Rhabditida	Rhabditidae	1	1	1	0	0	1	1
Monhystera	Nematodes	Chromad orea	Monhysterida	Monhysteridae	1	1	1	0	0	1	1
Rhabditophanes	Nematodes	Chromad orea	Rhabditida	Alloionematidae	1	1	1	0	0	1	1
Rhabditis	Nematodes	Chromad orea	Rhabditida	Rhabditidae	1	1	1	0	0	1	1
Pelodera	Nematodes	Chromad orea	Rhabditida	Rhabditidae	1	1	1	0	0	1	1
Aphelenchoides	Nematodes	Chromad orea	Rhabditida	Aphelenchoididae	1	1	1	0	0	1	1
Ditylenchus	Nematodes	Chromad orea	Rhabditida	Anguinidae	1	1	1	0	0	1	1
Filenchus	Nematodes	Chromad orea	Rhabditida	Tylenchidae	1	1	1	0	0	1	1
Aphelenchus	Nematodes	Chromad orea	Rhabditida	Aphelenchidae	1	1	1	0	0	1	1
Diphtherophora	Nematodes	Enoplea	Triplonchida	Diphtherophoridae	1	1	1	0	0	1	1
Tylencholaimus	Nematodes	Enoplea	Dorylaimida	Tylencholaimidae	1	1	1	0	0	1	1
Tylolaimophorus	Nematodes	Enoplea	Triplonchida	Diphtherophoridae	1	1	1	0	0	1	1
Tylencholaimellus	Nematodes	Enoplea	Dorylaimida	Tylencholaimellida e	1	1	1	0	0	1	1
Ecphyadophora	Nematodes	Chromad orea	Rhabditida	Ecphyadophoridae	1	1	1	0	0	1	1
Lelenchus	Nematodes	Chromad orea	Rhabditida	Ecphyadophoridae	1	1	1	0	0	1	1
Tylenchus	Nematodes	Chromad orea	Rhabditida	Tylenchidae	1	1	1	0	0	1	1
Paratylenchus	Nematodes	Chromad orea	Rhabditida	Tylenchulidae	1	1	1	0	0	1	1
Malenchus	Nematodes	Chromad orea	Rhabditida	Tylenchidae	1	1	1	0	0	1	1
Meloidogyne	Nematodes	Chromad orea	Rhabditida	Meloidogynidae	1	1	1	0	0	1	1
Pratylenchus	Nematodes	Chromad orea	Rhabditida	Pratylenchidae	1	1	1	0	0	1	1
Miculenchus	Nematodes	Chromad orea	Rhabditida	Tylenchidae	1	1	1	0	0	1	1
Boleodorus	Nematodes	Chromad orea	Rhabditida	Tylenchidae	1	1	1	0	0	1	1
Cephalenchus	Nematodes	Chromad orea	Rhabditida	Tylenchidae	1	1	1	0	0	1	1
Coslenchus	Nematodes	Chromad orea	Rhabditida	Tylenchidae	1	1	1	0	0	1	1

Zygotylenchus	Nematodes	Chromadorea	Rhabditida	Pratylenchidae	1	1	1	0	0	1	1
Basiria	Nematodes	Chromadorea	Rhabditida	Tylenchidae	1	1	1	0	0	1	1
Psilenchus	Nematodes	Chromadorea	Rhabditida	Tylenchidae	1	1	1	0	0	1	1
Tylenchorhynchus	Nematodes	Chromadorea	Rhabditida	Telotylenchidae	1	1	1	0	0	1	1
Neopsilenchus	Nematodes	Chromadorea	Rhabditida	Tylenchidae	1	1	1	0	0	1	1
Hoplotylus	Nematodes	Chromadorea	Rhabditida	Pratylenchidae	1	1	1	0	0	1	1
Helicotylenchus	Nematodes	Chromadorea	Rhabditida	Hoplolaimidae	1	1	1	0	0	1	1
Trichodorus	Nematodes	Enoplea	Triplonchida	Trichodoridae	1	1	1	0	0	1	1
Merlinius	Nematodes	Chromadorea	Rhabditida	Merliniidae	1	1	1	0	0	1	1
Dorylaimellus	Nematodes	Enoplea	Dorylaimida	Belondiridae	1	1	1	0	0	1	1
Paratrichodorus	Nematodes	Enoplea	Triplonchida	Trichodoridae	1	1	1	0	0	1	1
Paratrophurus	Nematodes	Chromadorea	Rhabditida	Telotylenchidae	1	1	1	0	0	1	1
Hemicriconemoides	Nematodes	Chromadorea	Rhabditida	Criconematidae	1	1	1	0	0	1	1
Rotylenchus	Nematodes	Chromadorea	Rhabditida	Hoplolaimidae	1	1	1	0	0	1	1
Bitylenchus	Nematodes	Chromadorea	Rhabditida	Telotylenchidae	1	1	1	0	0	1	1
Mesocriconema	Nematodes	Chromadorea	Rhabditida	Criconematidae	1	1	1	0	0	1	1
Geocenamus	Nematodes	Chromadorea	Rhabditida	Merliniidae	1	1	1	0	0	1	1
Longidorella	Nematodes	Enoplea	Dorylaimida	Nordiidae	1	1	1	0	0	1	1
Criconema	Nematodes	Chromadorea	Rhabditida	Criconematidae	1	1	1	0	0	1	1
Criconemoides	Nematodes	Chromadorea	Rhabditida	Criconematidae	1	1	1	0	0	1	1
Hemicycliophora	Nematodes	Chromadorea	Rhabditida	Hemicycliophoridae	1	1	1	0	0	1	1
Rotylenchulus	Nematodes	Chromadorea	Rhabditida	Hoplolaimidae	1	1	1	0	0	1	1
Axonchium	Nematodes	Enoplea	Dorylaimida	Belondiridae	1	1	1	0	0	1	1
Longidorus	Nematodes	Enoplea	Dorylaimida	Longidoridae	1	1	1	0	0	1	1
Xiphinema	Nematodes	Enoplea	Dorylaimida	Longidoridae	1	1	1	0	0	1	1
Microdorylaimus	Nematodes	Enoplea	Dorylaimida	Qudsianematidae	1	1	1	0	0	1	1
Campydora	Nematodes	Enoplea	Enoplida	Campydoridae	1	1	1	0	0	1	1
Dorydorella	Nematodes	Enoplea	Dorylaimida	Nordiidae	1	1	1	0	0	1	1

Dorylaimoides	Nematodes	Enoplea	Dorylaimida	Mydonomidae	1	1	1	0	0	1	1
Prodorylaimus	Nematodes	Enoplea	Dorylaimida	Dorylaimidae	1	1	1	0	0	1	1
Ecumenicus	Nematodes	Enoplea	Dorylaimida	Qudsianematidae	1	1	1	0	0	1	1
Discolaimium	Nematodes	Enoplea	Dorylaimida	Qudsianematidae	1	1	1	0	0	1	1
Epidorylaimus	Nematodes	Enoplea	Dorylaimida	Qudsianematidae	1	1	1	0	0	1	1
Pungentus	Nematodes	Enoplea	Dorylaimida	Nordiidae	1	1	1	0	0	1	1
Chrysonema	Nematodes	Enoplea	Dorylaimida	Qudsianematidae	1	1	1	0	0	1	1
Thornia	Nematodes	Enoplea	Dorylaimida	Qudsianematidae	1	1	1	0	0	1	1
Mesodorylaimus	Nematodes	Enoplea	Dorylaimida	Dorylaimidae	1	1	1	0	0	1	1
Eudorylaimus	Nematodes	Enoplea	Dorylaimida	Qudsianematidae	1	1	1	0	0	1	1
Discolaimus	Nematodes	Enoplea	Dorylaimida	Qudsianematidae	1	1	1	0	0	1	1
Enchodelus	Nematodes	Enoplea	Dorylaimida	Nordiidae	1	1	1	0	0	1	1
Allodorylaimus	Nematodes	Enoplea	Dorylaimida	Qudsianematidae	1	1	1	0	0	1	1
Aporcelaimellus	Nematodes	Enoplea	Dorylaimida	Aporcelaimidae	1	1	1	0	0	1	1
Paraxonchium	Nematodes	Enoplea	Dorylaimida	Aporcelaimidae	1	1	1	0	0	1	1
Laimydorus	Nematodes	Enoplea	Dorylaimida	Dorylaimidae	1	1	1	0	0	1	1
Sectonema	Nematodes	Enoplea	Dorylaimida	Aporcelaimidae	1	1	1	0	0	1	1
Aporcelaimus	Nematodes	Enoplea	Dorylaimida	Aporcelaimidae	1	1	1	0	0	1	1
Achromadora	Nematodes	Chromad orea	Chromadorida	Cyatholaimidae	1	1	1	0	0	1	1
Mylonchulus	Nematodes	Enoplea	Mononchida	Mylonchulidae	1	1	1	0	0	1	1
Tripyla	Nematodes	Enoplea	Triplonchida	Tripylidae	1	1	1	0	0	1	1
Mononchus	Nematodes	Enoplea	Mononchida	Mononchidae	1	1	1	0	0	1	1
Clarkus	Nematodes	Enoplea	Mononchida	Mononchidae	1	1	1	0	0	1	1
Oxydirus	Nematodes	Enoplea	Dorylaimida	Belondiridae	1	1	1	0	0	1	1
Thonus	Nematodes	Enoplea	Dorylaimida	Qudsianematidae	1	1	1	0	0	1	1
Anatonchus	Nematodes	Enoplea	Mononchida	Anatonchidae	1	1	1	0	0	1	1
Miconchus	Nematodes	Enoplea	Mononchida	Anatonchidae	1	1	1	0	0	1	1
Nygolaimus	Nematodes	Enoplea	Dorylaimida	Nygolaimidae	1	1	1	0	0	1	1
Coomansus	Nematodes	Enoplea	Mononchida	Mononchidae	1	1	1	0	0	1	1
Granonchulus	Nematodes	Enoplea	Mononchida	Mononchidae	1	1	1	0	0	1	1
Labronema	Nematodes	Enoplea	Dorylaimida	Qudsianematidae	1	1	1	0	0	1	1
Prionchulus	Nematodes	Enoplea	Mononchida	Mononchidae	1	1	1	0	0	1	1
Blattisociidae	mesofauna	Arachnid a	Mesostigmata	Blattisociidae	1	0.7	1	0	0	1	0. 5
Laelapidae	mesofauna	Arachnid a	Mesostigmata	Laelapidae	1	0.7	1	0	0	1	0. 5

Macrochelidae	mesofauna	Arachnid a	Mesostigmata	Macrochelidae	1	0.7	1	0	0	1	0. 5
Parasitidae	mesofauna	Arachnid a	Mesostigmata	Parasitidae	1	0.7	1	0	0	1	0. 5
Rhodacaridae	mesofauna	Arachnid a	Mesostigmata	Rhodacaridae	1	0.7	1	0	0	1	0. 5
Veigaiidae	mesofauna	Arachnid a	Mesostigmata	Veigaiidae	1	0.7	1	0	0	1	0. 5
Gamasina_juv	mesofauna	Arachnid a	Mesostigmata	NA	1	0.7	1	0	0	1	0. 5
Acaridae	mesofauna	Arachnid a	Sarcoptiformes	Acaridae	1	1	1	0	0	1	0. 5
Astegistidae	mesofauna	Arachnid a	Sarcoptiformes	Astegistidae	1	0.4	0.7	0	0	1	0. 5
Autognethidae	mesofauna	Arachnid a	Sarcoptiformes	Autognethidae	1	0.4	0.7	0	0	1	0. 5
Brachychthoniidae	mesofauna	Arachnid a	Sarcoptiformes	Brachychthoniidae	1	0.4	0.7	0	0	1	0. 5
Ceratozetidae	mesofauna	Arachnid a	Sarcoptiformes	Ceratozetidae	1	0.4	0.7	0	0	1	0. 5
Euphthiracaridae	mesofauna	Arachnid a	Sarcoptiformes	Euphthiracaridae	1	0.4	0.7	0	0	1	0. 5
Hypochthoniidae	mesofauna	Arachnid a	Sarcoptiformes	Hypochthoniidae	1	0.4	0.7	0	0	1	0. 5
Oppiidae	mesofauna	Arachnid a	Sarcoptiformes	Oppiidae	1	0.4	0.7	0	0	1	0. 5
Oribatulidae	mesofauna	Arachnid a	Sarcoptiformes	Oribatulidae	1	0.4	0.7	0	0	1	0. 5
Protoribatidae	mesofauna	Arachnid a	Sarcoptiformes	Protoribatidae	1	0.4	0.7	0	0	1	0. 5
Scheloribatidae	mesofauna	Arachnid a	Sarcoptiformes	Scheloribatidae	1	0.4	0.7	0	0	1	0. 5
Tectocepheidae	mesofauna	Arachnid a	Sarcoptiformes	Tectocepheidae	1	0.4	0.7	0	0	1	0. 5
Oribatida_juv	mesofauna	Arachnid a	Sarcoptiformes	NA	1	0.4	0.7	0	0	1	0. 5
Eupodidae	mesofauna	Arachnid a	Trombidiformes	Eupodidae	1	0.7	1	0	0.5	1	0. 5
Penthaleidae	mesofauna	Arachnid a	Trombidiformes	Penthaleidae	1	0.7	1	0	0.5	1	0. 5
Pygmephoridae	mesofauna	Arachnid a	Trombidiformes	Pygmephoridae	1	0.7	1	0	0.5	1	0. 5
Rhagidiidae	mesofauna	Arachnid a	Trombidiformes	Rhagidiidae	1	0.7	1	0	0.5	1	0. 5
Scutacaridae	mesofauna	Arachnid a	Trombidiformes	Scutacaridae	1	0.7	1	0	0.5	1	0. 5
Stigmaeidae	mesofauna	Arachnid a	Trombidiformes	Stigmaeidae	1	0.7	1	0	0.5	1	0. 5
Tydaeidae	mesofauna	Arachnid a	Trombidiformes	Tydaeidae	1	0.7	1	0	0.5	1	0. 5

Prostigmata_juv_undet	mesofauna	Arachnida	Trombidiformes	NA	1	0.7	1	0	0.5	1	0.5
Entomobryidae_large	mesofauna	Collembola	Entomobryomorpha	Entomobryidae	0.7	1	1	0.5	1	0.5	0
Entomobryidae_medium	mesofauna	Collembola	Entomobryomorpha	Entomobryidae	0.7	1	1	0.5	1	0.5	0
Isotomidae_large	mesofauna	Collembola	Entomobryomorpha	Isotomidae	0.7	1	1	0.5	1	0.5	0
Isotomidae_medium	mesofauna	Collembola	Entomobryomorpha	Isotomidae	0.7	1	1	0	0	1	0.5
Isotomidae_small	mesofauna	Collembola	Entomobryomorpha	Isotomidae	0.7	1	1	0	0	1	0.5
Neelidae	mesofauna	Collembola	Neelipleona	Neelidae	0.7	1	1	0	0	1	0.5
Hypogastruridae	mesofauna	Collembola	Poduromorpha	Hypogastruridae	0.7	1	1	0	1	1	1
Onychiuridae	mesofauna	Collembola	Poduromorpha	Onychiuridae	1	1	0.4	0	0	1	1
Tullbergiidae	mesofauna	Collembola	Poduromorpha	Tullbergiidae	1	1	0.4	0	0	1	1
Arrhopalitidae	mesofauna	Collembola	Symphypleona	Arrhopalitidae	0.7	1	1	0	0	1	0.5
Katiannidae	mesofauna	Collembola	Symphypleona	Katiannidae	0.7	1	1	0.5	1	0.5	0
Sminthurididae	mesofauna	Collembola	Symphypleona	Sminthurididae	0.7	1	1	0.5	1	0.5	0
Pauropoda	mesofauna	Pauropoda	Tetramerocerata	Pauropodidae	1	1	1	0	0	1	1
Protura	mesofauna	Protura	Protura	Eosentomidae	1	1	1	0	0	1	1
Symphyla	mesofauna	Symphyla	Symphyla	NA	1	1	0.4	0	0	1	1
Linyphiidae_ad	macrofauna	Arachnida	Araneae	Linyphiidae	1	1	1	0	1	0.5	0
Linyphiidae_juv	macrofauna	Arachnida	Araneae	Linyphiidae	1	1	1	0	0.5	1	0
Geophilidae	macrofauna	Chilopoda	Geophilomorpha	Geophilidae	1	1	1	0	0	1	1
Schendylidae	macrofauna	Chilopoda	Geophilomorpha	Schendylidae	1	1	1	0	0	1	1
Lithobiidae_large	macrofauna	Chilopoda	Lithobiomorpha	Lithobiidae	1	1	1	0	0.5	1	0
Lithobiidae_small	macrofauna	Chilopoda	Lithobiomorpha	Lithobiidae	1	1	1	0	0.5	1	0
Blaniulidae_large	macrofauna	Diplopoda	Julida	Blaniulidae	1	0.4	0.7	0	1	1	0.5
Blaniulidae_small	macrofauna	Diplopoda	Julida	Blaniulidae	1	0.4	0.7	0	1	1	0.5
Julidae	macrofauna	Diplopoda	Julida	Julidae	1	0.4	0.7	0	1	1	0.5
Polydesmidae_large	macrofauna	Diplopoda	Polydesmida	Polydesmidae	1	0.4	0.7	0	1	1	0
Polydesmidae_small	macrofauna	Diplopoda	Polydesmida	Polydesmidae	1	0.4	0.7	0	1	1	0

Campodeidae	macrofauna	Diplura	Diplura	Campodeidae	1	1	1	0	0	1	1
Ferussaciidae	macrofauna	Gastropoda	Stylommatophora	Ferussaciidae	1	0.4	0.4	0.5	1	1	0
Valloniidae	macrofauna	Gastropoda	Stylommatophora	Valloniidae	1	0.4	0.4	0.5	1	1	0
Curculionidae_ad	macrofauna	Insecta	Coleoptera	Curculionidae	1	0.4	1	0	0	1	1
Elateridae_larva	macrofauna	Insecta	Coleoptera	Elateridae	1	0.4	1	0	0	1	1
Staphylinidae_ad	macrofauna	Insecta	Coleoptera	Staphylinidae	1	0.4	0.4	1	1	1	1
Chironomidae_ad	macrofauna	Insecta	Diptera	Chironomidae	1	1	1	1	0.5	1	0
Nematocera_larva	macrofauna	Insecta	Diptera	NA	1	1	1	1	1	1	0
Aphididae	macrofauna	Insecta	Hemiptera	Aphididae	1	0.7	0.7	0	1	1	1
Coccidae	macrofauna	Insecta	Hemiptera	Coccidae	1	0.7	0.7	0	1	1	1

Supplementary References

1. Li, J. *et al.* A size-constrained feeding-niche model distinguishes predation patterns between aquatic and terrestrial food webs. *Ecology Letters* ele.14134 (2022) doi:10.1111/ele.14134.
2. Potapov, A. M. Multifunctionality of belowground food webs: resource, size and spatial energy channels. Biological Reviews brv.12857 (2022) doi:10.1111/brv.12857.
