# Supplementary material for: Soil community history strengthens belowground multitrophic functioning across plant diversity levels in a grassland experiment
Source: Nat Commun. 2024 Nov 19;15:10029. doi: 10.1038/s41467-024-54401-z (PMC11577027; doi:10.1038/s41467-024-54401-z)
Supplement: Supplementary file 2 — Reporting Summary [file 41467_2024_54401_MOESM2_ESM.pdf]

Corresponding author(s): Angelos Amyntas

Last updated by author(s): Oct 25, 2024

Reporting Summary

Nature Portfolio wishes to improve the reproducibility of the work that we publish. This form provides structure for consistency and transparency in reporting. For further information on Nature Portfolio policies, see our Editorial Policies and the Editorial Policy Checklist.

Statistics

For all statistical analyses, confirm that the following items are present in the figure legend, table legend, main text, or Methods section.

n/a	Confirmed	
☐	☒	The exact sample size (*n*) for each experimental group/condition, given as a discrete number and unit of measurement
☐	☒	A statement on whether measurements were taken from distinct samples or whether the same sample was measured repeatedly
☒	☐	The statistical test(s) used AND whether they are one- or two-sided<br>*Only common tests should be described solely by name; describe more complex techniques in the Methods section.*
☐	☒	A description of all covariates tested
☐	☒	A description of any assumptions or corrections, such as tests of normality and adjustment for multiple comparisons
☐	☒	A full description of the statistical parameters including central tendency (e.g. means) or other basic estimates (e.g. regression coefficient) AND variation (e.g. standard deviation) or associated estimates of uncertainty (e.g. confidence intervals)
☒	☐	For null hypothesis testing, the test statistic (e.g. *F*, *t*, *r*) with confidence intervals, effect sizes, degrees of freedom and *P* value noted<br>*Give P values as exact values whenever suitable.*
☐	☒	For Bayesian analysis, information on the choice of priors and Markov chain Monte Carlo settings
☐	☒	For hierarchical and complex designs, identification of the appropriate level for tests and full reporting of outcomes
☒	☐	Estimates of effect sizes (e.g. Cohen's *d*, Pearson's *r*), indicating how they were calculated

Our web collection on statistics for biologists contains articles on many of the points above.

Software and code

Policy information about availability of computer code

Data collection	No software was used for data collection
Data analysis	Code is available in the GitHub repository https://github.com/amynang/jenatron_soilfoodwebs and archived in Zenodo (https://doi.org/10.5281/zenodo.13923794).

For manuscripts utilizing custom algorithms or software that are central to the research but not yet described in published literature, software must be made available to editors and reviewers. We strongly encourage code deposition in a community repository (e.g. GitHub). See the Nature Portfolio guidelines for submitting code & software for further information.

Data

Policy information about availability of data

All manuscripts must include a data availability statement. This statement should provide the following information, where applicable:

- Accession codes, unique identifiers, or web links for publicly available datasets
- A description of any restrictions on data availability
- For clinical datasets or third party data, please ensure that the statement adheres to our policy

Data are available in the GitHub repository https://github.com/amynang/jenatron_soilfoodwebs and archived in Zenodo (https://doi.org/10.5281/zenodo.13923794).

Research involving human participants, their data, or biological material

Policy information about studies with human participants or human data. See also policy information about sex, gender (identity/presentation), and sexual orientation and race, ethnicity and racism.

Reporting on sex and gender	*Use the terms sex (biological attribute) and gender (shaped by social and cultural circumstances) carefully in order to avoid confusing both terms. Indicate if findings apply to only one sex or gender; describe whether sex and gender were considered in study design; whether sex and/or gender was determined based on self-reporting or assigned and methods used. Provide in the source data disaggregated sex and gender data, where this information has been collected, and if consent has been obtained for sharing of individual-level data; provide overall numbers in this Reporting Summary. Please state if this information has not been collected. Report sex- and gender-based analyses where performed, justify reasons for lack of sex- and gender-based analysis.*
Reporting on race, ethnicity, or other socially relevant groupings	*Please specify the socially constructed or socially relevant categorization variable(s) used in your manuscript and explain why they were used. Please note that such variables should not be used as proxies for other socially constructed/relevant variables (for example, race or ethnicity should not be used as a proxy for socioeconomic status). Provide clear definitions of the relevant terms used, how they were provided (by the participants/respondents, the researchers, or third parties), and the method(s) used to classify people into the different categories (e.g. self-report, census or administrative data, social media data, etc.) Please provide details about how you controlled for confounding variables in your analyses.*
Population characteristics	*Describe the covariate-relevant population characteristics of the human research participants (e.g. age, genotypic information, past and current diagnosis and treatment categories). If you filled out the behavioural & social sciences study design questions and have nothing to add here, write "See above."*
Recruitment	*Describe how participants were recruited. Outline any potential self-selection bias or other biases that may be present and how these are likely to impact results.*
Ethics oversight	*Identify the organization(s) that approved the study protocol.*

Note that full information on the approval of the study protocol must also be provided in the manuscript.

Field-specific reporting

Please select the one below that is the best fit for your research. If you are not sure, read the appropriate sections before making your selection.

☐ Life sciences ☐ Behavioural & social sciences ☒ Ecological, evolutionary & environmental sciences

For a reference copy of the document with all sections, see nature.com/documents/nr-reporting-summary-flat.pdf

Ecological, evolutionary & environmental sciences study design

All studies must disclose on these points even when the disclosure is negative.

Study description	The aim of the study was to assess the dependence of soil fauna community trophic functioning on plant diversity, soil history and plant history. This was conducted in a mesocosm experiment. It consisted of 24 EcoUnits, each containing four mesocosms. Mesocosms in each EcoUnit had the same plant species richness (either 1,2,3 or 6 species) and community-specific or unrelated soil history, crossed with community-specific or unrelated plant history.
Research sample	Soil invertebrates (nematodes, mesofauna, macrofauna) were extracted from soil cores taken from each of 96 mesocosms
Sampling strategy	At the end of the experiment, from every mesocosm we extracted one 15cm diameter soil core (for macrofauna), one 5cm diam. core (for mesofauna) and three 2cm diam. cores for nematodes. All cores were to 10cm depth. Macro and mesofauna were extracted using heat extraction. Nematodes were extracted from 20g of soil (after pooling the three 2cm cores), using the Baermann-funnel method.
Data collection	Macro and mesofauna identification to Family level and bodysize measurments were were conducted by Bernhard Klarner. Nematode identification was conducted by Krassimira Ilieva-Makulec. Body size information of nematode taxa was retrived from Nemaplex.
Timing and spatial scale	Sampling took place four months after the establishment of the mesocosm communities, in October 22-28 2022
Data exclusions	Data from all 96 mesocosms were included in the analysis
Reproducibility	All code necessary to reproduce the analysis is deposited in GutHub and archived in Zenodo. (see above)
Randomization	The arrangement of the four soil-plant history treatments in each EcoUnit was random. Ecounits of different plant diversity were randomly positioned within six spatial blocks.

Blinding	During sample processing, samples were assigned alphanumeric names to indicate Ecounit and subunit with no indication of plant richness or history treatment of that unit/subunit.

Did the study involve field work? ☐ Yes ☒ No

Reporting for specific materials, systems and methods

We require information from authors about some types of materials, experimental systems and methods used in many studies. Here, indicate whether each material, system or method listed is relevant to your study. If you are not sure if a list item applies to your research, read the appropriate section before selecting a response.

Materials & experimental systems

n/a	Involved in the study
☒	☐ Antibodies
☒	☐ Eukaryotic cell lines
☒	☐ Palaeontology and archaeology
☐	☒ Animals and other organisms
☒	☐ Clinical data
☒	☐ Dual use research of concern
☐	☒ Plants

Methods

n/a	Involved in the study
☒	☐ ChIP-seq
☒	☐ Flow cytometry
☒	☐ MRI-based neuroimaging

Animals and other research organisms

Policy information about studies involving animals; ARRIVE guidelines recommended for reporting animal research, and Sex and Gender in Research

Laboratory animals	The study did not involve laboratory animals.
Wild animals	The study involved soil invertebrates, which at the end of the experiment were extracted from soil originally coming from experimental plots in a long-term field experiment. The method of extraction kills the animals which is also necessary for their identification.
Reporting on sex	The study was examining community level properties and processes of the soil invertebrate community. Sex-based analysis was not relevant.
Field-collected samples	The experiment was conducted in mesocosms. For each mesocosm, a precipitation regime was set at 450 ml per 12 h. A light regime was established with a 16/8 h day-night cycle, including one-hour dusk and dawn at 25 % light intensity (100 % ~ 370 µmol/m²/s). To simulate natural seasonality, the day lengths were shortened from August on in full-hour steps.
Ethics oversight	No ethical approval was required.

Note that full information on the approval of the study protocol must also be provided in the manuscript.

Dual use research of concern

Policy information about dual use research of concern

Hazards

Could the accidental, deliberate or reckless misuse of agents or technologies generated in the work, or the application of information presented in the manuscript, pose a threat to:

No	Yes
☒	☐ Public health
☒	☐ National security
☒	☐ Crops and/or livestock
☒	☐ Ecosystems
☒	☐ Any other significant area

Experiments of concern

Does the work involve any of these experiments of concern:

No	Yes	
☒	☐	Demonstrate how to render a vaccine ineffective
☒	☐	Confer resistance to therapeutically useful antibiotics or antiviral agents
☒	☐	Enhance the virulence of a pathogen or render a nonpathogen virulent
☒	☐	Increase transmissibility of a pathogen
☒	☐	Alter the host range of a pathogen
☒	☐	Enable evasion of diagnostic/detection modalities
☒	☐	Enable the weaponization of a biological agent or toxin
☒	☐	Any other potentially harmful combination of experiments and agents

Plants

Seed stocks	Plant communities in mesocosms were assembled by transplanting seedlings grown in a greenhouse. For the mesocosms with community-specific plant history, seeds came from the reference experimental community in the Trait-Based Experiment, in the Jena Experiment. For the mesocosms without such history seeds came from the original seed material used to establish the Trait-Based Experiment in 2010.
Novel plant genotypes	Not applicable.
Authentication	Not applicable.
