# Supplementary material for: Soil community history strengthens belowground multitrophic functioning across plant diversity levels in a grassland experiment
Source: Nat Commun. 2024 Nov 19;15:10029. doi: 10.1038/s41467-024-54401-z (PMC11577027; doi:10.1038/s41467-024-54401-z)
Supplement: Supplementary file 3 — Transparent Peer Review file [file 41467_2024_54401_MOESM3_ESM.pdf]

nature portfolio

Peer Review File

Soil community history strengthens belowground multitrophic functioning across plant diversity levels in a grassland experiment

Corresponding Author: Angelos Amyntas

This file contains all reviewer reports in order by version, followed by all author rebuttals in order by version.

Version 0:

Reviewer comments:

Reviewer #1

(Remarks to the Author)
I read the manuscript by Amyntas et al. with great interest. They use a mesocosm study started from soil monoliths taken from the Jena experiment to test the effects of plant diversity on the composition of the soil community. Then they use a common food web model (fluxweb) to calculate the energy flux through these communities relying on several well-documented assumptions about diet, physiology, and community equilibrium.
I have four important questions that I hope the authors can address and find helpful.
First, the authors apply a food web energy flux model that assumes a stable equilibrium, but their experimental data come from mesocosms that are unlikely to meet this assumption. By this, I do not mean to invoke the common critique of these models that ‘no ecological system is at equilibrium’. For example, the Jena experiment has been run long enough that I think the authors could have made a compelling argument that those communities may be close enough to a new equilibrium, so that the error of estimating equilibrium is small. Even in this case, I would recommend a sensitivity analysis (see comment 2). However, in this study, the authors removed monoliths and established them in a new environment creating a shift in environmental conditions, some amount of freshly killed root material beginning to decompose, and new edge effects along the end of the core. They were then planted with new seedlings, which were in the process of growing during the experiment. Finally, the experiment only lasted 5-months. All these factors make me concerned that the final community measurements may be far from an equilibrium. If this is the case, then the model assumption of equilibrium is invalid. The authors can address this comment by demonstrating that their high-level results do not change if they relax the assumption of equilibrium. This could be done by simulating alternative biomass levels that are within a plausible range of equilibrium. They could also use the technique proposed by de Ruiter et al. 1993 in Journal of Applied Ecology whereby change over time is used. This is Eqn 4 in that manuscript. They could apply this technique if they have baseline data about the abundance of different groups in the Jena Experimental plots, so that change over 5-months is final abundance minus baseline abundance.
Second and related to the first comment, the authors should conduct a sensitivity analysis of their fluxweb simulation. Built in functionality exists in fluxweb to run this analysis. Key parameters for sensitivity analysis would be (1) equilibrium biomass—using the error distribution/coefficient of variation estimated from empirical replicates, (2) metabolic rates, (3) loss rates. The authors should then verify that there overall results are robust to these sensitivity analyses.
Third, Figure 4 shows the relationships between community weighted mean body mass and total community biomass against community level energy flux. As the authors rightly pointed out in the paragraph on line 151, these are expected relationships. In fact, they are a consequence of the model structural assumptions whereby flux rate is determined by overall losses, which are themselves linked to biomass and body mass by the metabolic scaling equations used. I suggest that the authors modify this presentation to make it much clearer that these relationships are based largely on the model assumptions and are not results of a new or unexpected analysis. This could be achieved by moving this to the methods section or putting the Figure in the supplementary material. The current presentation as the final figure of the manuscript detracts in my opinion from the main findings in Figures 2 and 3.
Finally, I suggest that the authors remove the term multitrophic functioning from the title and replace it with energy flux there and throughout the manuscript. Multitrophic functioning in my opinion is opaque and technical—it could mean many different measurements and seems to imply that the authors measured many ways that food web functions changed. Energy flux on the other hand is exactly what was modelling, and the result presented other than diversity and biomass. Energy flux through a food web is also a much more intuitive concept for non-experts.

Line comments:
Line 235: I found that this paragraph was largely speculative and outside the scope of the project, since community assembly from outside the mesocosms would not have occurred in the experiment.
Line 251: accumulating instead of adding evidence.

Reviewer #2

(Remarks to the Author)
This paper addresses a significant scientific challenge: understanding the drivers of ecosystem functioning across multiple trophic levels and of their underlying mechanisms. This study is particularly valuable because it focuses on the soil food webs (of soil fauna) — an area often overlooked in previous studies. It goes beyond simply examining the trophic composition of communities by diving into community energy dynamics. The authors achieved this by using energy fluxes of the entire food web and of each trophic level as the proxies of the multitrophic ecosystem functioning. One of the key strengths of this study is the author's' ability to experimentally disentangle the effects of plant diversity, soil history and plant history on multitrophic ecosystem functioning. To my knowledge this is the first study that address the importance of mechanisms in the field of food-web energetics.

The methodology used is sound, the work meets the expected standards in the field. The dataset used to address the research questions is relevant and presented exceptionally well, supported by robust statistical analyses. The high level of data collection and analysis makes this study the state-of-art within the established literature in this field.

The results are noteworthy, showing that soil community history governs the multitrophic function of soil food web. These effects are mediated by the altered community biomass and through the shift in community toward smaller-bodied taxa. Interesting, that the effects of plant diversity and of plant history were not significant, but the results suggest that the shift in community composition can be a stronger driver of ecosystem functioning than the effects of plant species number per se.

The figures are generally self-explanatory, although there are a few minor exceptions (see specific comments below). The ideas presented are well-structured, although there are some cases where sentences and phrases are too complex. Breaking them down into simpler parts would enhance clarity. The discussion section provides reasonable evidence to explain the obtained results, although the clarity could be improved in a several cases (see specific comments below). The work supports the conclusions and claims, no additional evidence are required.

Specific comments

Abstract

Overall, the abstract effectively communicates the research topic and findings, but there are few places where it is not clear what the authors mean without looking through the manuscript, specifically:

Line 29-30: "…shared or unrelated plant-soil history."

Line 31: "…food-web energetics approach." Here I miss some more information what exactly the proxy of the trophic functioning is/are. I suggest replacing the "food-web energetics approach" by more specific information, e.g. "by assessing energy fluxes and biomass stocks across the food web"

Line 33: "..via biomass and body mass changes" It is not clear what do you mean by biomass here, is it the whole-community biomass? If so, isn't the biomass also a proxy of the multitrophic function? Please clarify.

Line 34: "Soil community history significantly influenced belowground multitrophic function via biomass and body mass changes, while plant diversity and history had unclear effects." I don't understand what "history" means in the second part of the sentence, given that the soil community history has being already mentioned in the first part of the sentence.

Line 35: "… importance of soil community assembly over time …" It is not clear what you mean by this sentence. Maybe change to "importance of the long-term community assembly processes" if this is what you mean by this sentence.

There are several complex phrases that make it difficult to understand the text. I suggest restructuring them as following:

Line 30: change "Soil fauna trophic functioning" to "The multitrophic functioning of soil fauna"

Line 36: "Disturbances disrupting soil community stability" to "Disturbances that disrupt soil community stability"

Introduction

Line 46: change "from" to "of"

Line 47: too many bracket, remove bracket before "e.g." and at the end of sentence

Line 49: change "complementarity" to "the complementarity effects"

Line 58-61: The sentence is difficult to understand because it includes a couple of complex structures, please rewrite or brake it down into few sentences

Line 57: The paragraph is too short and can be merged with the paragraph above

Line 65: Coma is missing before "such as" and before "is) in line 66

Line 70: Comas are missing before and after "in turn"

Line 72: Energy fluxes and carbon and nutrient cycles are themselves ecosystem functioning, please rephrase or explain what you mean by ecosystem functioning other than those two.

Line 74: add "plant related" before "functions"

Line 75: coma is missing before "such as"

Line 76: give the reference at the end of the statement

Line 80: replace "as plant and soil history" by "the history effects of plant and soil, "

Line 80-82: complex sentence, simplifying or restructuring it might improve readability

Line 86-90: very long and too complex sentence

Line 93: Not clear what you mean by multitrophic functions. I suggest to introduce it before, when you talk about energetics approach, e.g. at the end of the sentence in line 90.

Line 96: Not clear what is the y-axis in Figure 1 (right panel)

Results

Line 107: rephrase "of mesocosm soil fauna food-webs"

Figure 2: figure letters overlap with the title of y-axis

Line 114: add "plant" before "history"

Lines 120-126, and all below: Maybe you can add the table with all the means and 95% HPD, so that you do not have to state them all individually in the text

Figure 3: Why there are no letters (post-hoc) at the figure a? If no differences you still can identify it by using the same letters across all groups. Also, it is not clear what community biomass mean (figure c), is it plant biomass or is it soil fauna biomass. Until now it was not mentioned before. Already in the introduction section you have to explain more clearly what are the variables for the multitrophic functioning. Add legend to the figure.

Line 144: Which specific Supplement?

Figure 4: Add legend to the figure

Line 151: "These findings imply that assembly had been leading to".. Here, please be more specific what assembly processes, e.g. under the soil history effects ? or animal community assembly?

Line 154: add literature reference to the statement "due to a higher 153 metabolic rate of smaller organisms"

Lines 151-155: these sentences belong to discussion section rather than to results.

Discussion

Line 186-187: it is not clear what authors mean by the "differences in plant density and community composition". Difference

from what or in what? Across treatments? Or in effects of plant diversity vs plant community composition?

Line 194: replace “for” by “to”

Line 226: This statement is repeated in Result section (lines 153-154)

Lines 241-242: replace “γ and β animal as well as plant 241 diversity” by “γ and β diversity of both animal and plant communities”

Line 249: replace “animal community assembly time” by “the assembly time of animal community”

Line 253: add literature reference supporting this statement “Given that local diversity is not necessarily declining despite global biodiversity loss”

Lines 239-258: The concluding statement almost totally is dedicated to only the lack of biodiversity effects. Authors should also focus here on the effects of soil history, which were the only effects that they found.

Materials and Methods

Line 331: which specific Supplement are you referring to?

Line 362: There is no reference 82 in the reference list

Statistical analysis: it is not stated why the CWM and community biomass data were log transformed (as shown on Figure 3)

Fig. S1 in the supplementary file states that authors had two different calculations of energy fluxes, but I could not find the description of this in the method part. It would be great to show such uncertainty analysis, which would show if the results change if different approach is taken for the parameterization of food webs, for example not based on the average body-mass in mesocosm, but randomly sampled from the range of body-mass values in that mesocosm.

Reviewer #3

(Remarks to the Author)
This is an interesting study and I am quite intrigued by the incubation of full soil monoliths, and the estimation of soil fauna energy fluxes and ecological function at the community scale. I also agree that the question being addressed is exciting and important. My biggest concern with the study though relates to the concept of a "soil history" when it comes to interpreting the fauna data. For instance, at lines 158-160 the authors state that fauna-driven functioning is promoted in soils with a shared history with plant communities vs soil communities from bare ground (i.e. “no shared history”). I agree that there were clear and interesting differences observed in fauna functioning in soils collected from these two sites (TBE site vs bare soil site), but I do not believe the data support the idea that these differences result from the sites having vs not having a shared plant history. First, it seems very likely that the differences observed in soil fauna function were driven simply by the inherent differences in soil properties between the Trait-Based Experiment site and the bare ground site combined with any management practices used to generate and maintain the desired plant conditions (spp diversity combinations vs bare ground) at those sites. Second, it is stated in the discussion that the bare soil plots did in fact have plants that invaded the plots from field borders. So, in addition to being driven by inherent differences among these two soils, fauna function may also have been shaped by legacy differences in the plant inputs between the TBE and bare soil sites. In other words, given that the plant history treatments had little-to-no impact on fauna functon(neither within or across soil types), the authors should interpret their results less through the lens of “shared history” and should entertain other potential explanations for the differences in function between the TBE and bare soils. True, this is a bit messier than the concise soil-by-plant history framework, but I feel it is much more accurate to the data.
I am also not that surprised by the lack of plant history treatment effects given the plant treatments were only in place for 4-5 months although I see this is acknowledged by the authors in the discussion.

Additional comments:
Line 123 – reference to Fig 1c-e should be Fig 2c-e, correct?
Line 270 - how were the monoliths collected AND were they the same size as cited above (50cm diam x 80cm deep) for the inner lysimeter?

Line 272-275 - later it states that seeds from the TBE site (i.e. with a shared history) were planted into the monoliths, but were there already any existing plants when monoliths were collected in spring? Also, did any seedlings emerge during the study that were not planted at the onset of the experiment? If so, were they removed by hand?

Line 275-277 - How were the plots maintained bare for 20 years? In my experience, this typically requires something dramatic like herbicides but it is not explained in the Jena experiment paper referenced. Whatever the method, could there also be legacy effects of such practices on fauna communities unrelated to the introduced history treatments?

Line 286-288 - How was this maintained for the 5 month duration? Were unwanted plants removed by hand?

Results: While I acknowledge there isn’t room for this in the main text, I think it would be a great addition to the appendix to include a table of mean abundances for the fauna…some degree of taxon lumping would be fine given the number of taxa observed.

Version 1:

Reviewer comments:

Reviewer #3

(Remarks to the Author)
I appreciate the author's attention to comments on the first draft of this manuscript. They have taken the time to address my concerns thoroughly, including those related to concerns about level of detail in their methods section that led me to question their ability to separate plant and soil effects as well as any unintended effects stemming from field maintenance and the removal of weeds during the monolith study. I also appreciate their addition of the new fauna table in the appendix. I stand by my original assessment that the full soil monolith and energy flux modeling conducted here are very interesting and that they will be of significance to the field of soil ecology. I feel the manuscript is now suitable for publication in Nature Communications.

(Remarks on code availability)

Reviewer #4

(Remarks to the Author)
This manuscript takes advantage of the long-term JENA experiment, enabling the authors to cleverly separate the impacts of plant community history and soil history on ecosystem functioning mediated by soil fauna. The sampling methodology and the description of the energetic food web modeling are clearly articulated and executed well, demonstrating a significant amount of work, especially given the experiment's relatively short duration of just four months. The inclusion of a sensitivity analysis strengthens the overall analysis, supporting the main conclusion that soil community history enhances multitrophic functioning. The manuscript is well-written, with clear illustrations that enhance readability.

Overall, the authors have done an admirable job addressing the concerns raised by the initial reviewers, and their efforts should be recognized. This work will contribute meaningfully to our understanding of how soil fauna influence ecosystem functioning.

The one thing that I think could be addressed more effectively is how to introduce the term ‘multitrophic functioning.’ I’m okay with it being used in the title, but I agree with Reviewer 1 that this term is opaque without context, and the manuscript presents it as if it is a widely accepted term when only a handful of articles have ever used it. However, I do like the idea of using a term that succinctly captures the link between food webs and ecosystem functioning, so one suggestion would be to explicitly define this term early in the Introduction instead of assuming the reader would immediately know what it describes.

(Remarks on code availability)

Open Access This Peer Review File is licensed under a Creative Commons Attribution 4.0 International License, which permits use, sharing, adaptation, distribution and reproduction in any medium or format, as long as you give appropriate credit to the original author(s) and the source, provide a link to the Creative Commons license, and indicate if changes were made.
In cases where reviewers are anonymous, credit should be given to 'Anonymous Referee' and the source.
The images or other third party material in this Peer Review File are included in the article's Creative Commons license, unless indicated otherwise in a credit line to the material. If material is not included in the article's Creative Commons license and your intended use is not permitted by statutory regulation or exceeds the permitted use, you will need to obtain permission directly from the copyright holder.
To view a copy of this license, visit https://creativecommons.org/licenses/by/4.0/

REVIEWER COMMENTS

Reviewer #1 (Remarks to the Author):

I read the manuscript by Amyntas et al. with great interest. They use a mesocosm study started from soil monoliths taken from the Jena experiment to test the effects of plant diversity on the composition of the soil community. Then they use a common food web model (fluxweb) to calculate the energy flux through these communities relying on several well-documented assumptions about diet, physiology, and community equilibrium.

I have four important questions that I hope the authors can address and find helpful.

First, the authors apply a food web energy flux model that assumes a stable equilibrium, but their experimental data come from mesocosms that are unlikely to meet this assumption. By this, I do not mean to invoke the common critique of these models that 'no ecological system is at equilibrium'. For example, the Jena experiment has been run long enough that I think the authors could have made a compelling argument that those communities may be close enough to a new equilibrium, so that the error of estimating equilibrium is small. Even in this case, I would recommend a sensitivity analysis (see comment 2). However, in this study, the authors removed monoliths and established them in a new environment creating a shift in environmental conditions, some amount of freshly killed root material beginning to decompose, and new edge effects along the end of the core. They were then planted with new seedlings, which were in the process of growing during the experiment. Finally, the experiment only lasted 5-months. All these factors make me concerned that the final community measurements may be far from an equilibrium. If this is the case, then the model assumption of equilibrium is invalid. The authors can address this comment by demonstrating that their high-level results do not change if they relax the assumption of equilibrium. This could be done by simulating alternative biomass levels that are within a plausible range of equilibrium. They could also use the technique proposed by de Ruiter et al. 1993 in Journal of Applied Ecology whereby change over time is used. This is Eqn 4 in that manuscript. They could apply this technique if they have baseline data about the abundance of different groups in the Jena Experimental plots, so that change over 5-months is final abundance minus baseline abundance.

We appreciate the constructive suggestions on how to improve our manuscript!

We understand the reviewer's concern with regards to the equilibrium assumption. We are also grateful for their suggestions on how to address this. As we do not have baseline data that would allow us to follow the second option, we have instead followed the first option: We have conducted a sensitivity analysis by simulating variation in the observed densities of the soil fauna taxa as well as variation on metabolic rates. This way, we perturbed both population biomasses which also influence the interaction matrix but also population level losses (which are perturbed through both the densities and the individual rates). Importantly, adding some variation on the loss term is equivalent to relaxing this equilibrium assumption. While original gains have to compensate for metabolic rates, with this added term, gains have to compensate for metabolic rates plus an additional term that can simulate a loss (increase of the value for the loss term) or gain (decrease of the value for the loss term) of biomass per unit of time.

We found that our main findings are robust to these perturbations. This sensitivity analysis is detailed in section Supplementary Note 2 and Figure S2 of the supplemental material. We also report its results in lines 374-377 of the main text.

Second and related to the first comment, the authors should conduct a sensitivity analysis of their fluxweb simulation. Built in functionality exists in fluxweb to run this analysis. Key parameters for sensitivity analysis would be (1) equilibrium biomass—using the error distribution/coefficient of variation estimated from empirical replicates, (2) metabolic rates, (3) loss rates. The authors should then verify that there overall results are robust to these sensitivity analyses.

Thank you for this suggestion. Please see our response to the previous point.

Third, Figure 4 shows the relationships between community weighted mean body mass and total community biomass against community level energy flux. As the authors rightly pointed out in the paragraph on line 151, these are expected relationships. In fact, they are a consequence of the model structural assumptions whereby flux rate is determined by overall losses, which are themselves linked to biomass and body mass by the metabolic scaling equations used. I suggest that the authors modify this presentation to make it much clearer that these relationships are based largely on the model assumptions and are not results of a new or unexpected analysis. This could be achieved by moving this to the methods section or putting the Figure in the supplementary material. The current presentation as the final figure of the manuscript detracts in my opinion from the main findings in Figures 2 and 3.

We agree that Figure 4 could be misinterpreted as a finding rather than a well-understood relationship that helps explain the connection between the results shown in Figure 2 and those shown in Figure 3 (which was the intention of its inclusion). We have followed this suggestion and moved the figure to the supplementary material (Figure S1).

Finally, I suggest that the authors remove the term multitrophic functioning from the title and replace it with energy flux there and throughout the manuscript. Multitrophic functioning in my opinion is opaque and technical—it could mean many different measurements and seems to imply that the authors measured many ways that food web functions changed. Energy flux on the other hand is exactly what was modelling, and the result presented other than diversity and biomass. Energy flux through a food web is also a much more intuitive concept for non-experts.

Respectfully, we would like to push back on this suggestion. The functioning of multitrophic soil fauna communities and how it is shaped by plant diversity and community history is the main focus of this study. Using a food-web method to do this allows us to overcome limitations of other approaches, as we explain in lines 82-. In fact, we would argue that *energy flux* is the technical term here, while *(multi)trophic functioning* is what we are trying to quantify through the use of energy fluxes. This term is also strongly related to the concept of multifunctionality of ecological communities that is increasingly the focus of BEF research.

Line comments:

Line 235: I found that this paragraph was largely speculative and outside the scope of the project, since community assembly from outside the mesocosms would not have occurred in the experiment.

This paragraph is intended as a future directions section, emphasizing the importance of considering the landscape that surrounds a community and its influence on the composition and functioning of the latter. As the reviewer rightly points out, such processes cannot take place across isolated mesocosms. They are however at play in the field and even traditional BEF experiments have recently attempted to retrospectively adopt a metacommunity perspective (Furey et al., 2020 10.1002/ecy.3694). We therefore believe that future work needs to address this site-to-landscape interplay and its contribution to emerging BEF relationships. In the revised version of the manuscript, we make clear that this section is intended to inspire future research approaches [Lines 237-239].

Line 251: accumulating instead of adding evidence.

We have rephrased this sentence. lines [todo]

Reviewer #2 (Remarks to the Author):

This paper addresses a significant scientific challenge: understanding the drivers of ecosystem functioning across multiple trophic levels and of their underlying mechanisms. This study is particularly valuable because it focuses on the soil food webs (of soil fauna) – an area often overlooked in previous studies. It goes beyond simply examining the trophic composition of communities by diving into community energy dynamics. The authors achieved this by using energy fluxes of the entire food web and of each trophic level as the proxies of the multitrophic ecosystem functioning. One of the key strengths of this study is the author's' ability to experimentally disentangle the effects of plant diversity, soil history and plant history on multitrophic ecosystem functioning. To my knowledge this is the first study that address the importance of mechanisms in the field of food-web energetics.

The methodology used is sound, the work meets the expected standards in the field. The dataset used to address the research questions is relevant and presented exceptionally well, supported by robust statistical analyses. The high level of data collection and analysis makes this study the state-of-art within the established literature in this field.

The results are noteworthy, showing that soil community history governs the multitrophic function of soil food web. These effects are mediated by the altered community biomass and through the shift in community toward smaller-bodied taxa. Interesting, that the effects of plant diversity and of plant history were not significant, but the results suggest that the shift in community composition can be a stronger driver of ecosystem functioning than the effects of plant species number per se.

The figures are generally self-explanatory, although there are a few minor exceptions (see specific comments below). The ideas presented are well-structured, although there are some cases where sentences and phrases are too complex. Breaking them down into simpler parts would enhance clarity. The discussion section provides reasonable evidence to explain the obtained results, although the clarity could be improved in a several cases (see specific comments below). The work supports the conclusions and claims, no additional evidence are required.

We greatly appreciate this very positive feedback on our work as well as the thought and time that has been put into the following list of suggested improvements of our manuscript.

Specific comments

Abstract

Overall, the abstract effectively communicates the research topic and findings, but there are few places where it is not clear what the authors mean without looking through the manuscript, specifically:

Line 29-30: "…shared or unrelated plant-soil history."

We added a more detailed description to the abstract: "... we established experimental mesocosms replicating a gradient in plant species richness across treatments of shared vs. non shared history of (1) the plant community and (2) the soil fauna community."

Line 31: "…food-web energetics approach." Here I miss some more information what exactly the proxy of the trophic functioning is/are. I suggest replacing the "food-web energetics approach" by more specific information, e.g. "by assessing energy fluxes and biomass stocks across the food web"

We followed this suggestion.

Line 33: "..via biomass and body mass changes" It is not clear what do you mean by biomass here, is it the whole-community biomass? If so, isn't the biomass also a proxy of the multitrophic function? Please clarify.

In the revised version, we clarified this: "Soil community history significantly influenced belowground multitrophic function via changes in biomass stocks and the population-average body masses across the food webs …"

Line 34: "Soil community history significantly influenced belowground multitrophic function via biomass and body mass changes, while plant diversity and history had unclear effects." I don't understand what "history" means in the second part of the sentence, given that the soil community history has being already mentioned in the first part of the sentence.

We clarified that this point is related to plant history. In the revised version of the manuscript, we have split this statement into two sentences. "Soil community history significantly influenced belowground multitrophic function via changes in biomass stocks and community-average body mass changes across the food webs. However, variation in plant diversity and plant community history had unclear effects."

Line 35: "… importance of soil community assembly over time …" It is not clear what you mean by this sentence. Maybe change to "importance of the long-term community assembly processes" if this is what you mean by this sentence.

We followed this suggestion.

There are several complex phrases that make it difficult to understand the text. I suggest restructuring them as following:

Line 30: change “Soil fauna trophic functioning” to “The multitrophic functioning of soil fauna”

We followed this suggestion.

Line 36: “Disturbances disrupting soil community stability” to “Disturbances that disrupt soil community stability”

We have followed this suggestion.

Introduction

Line 46: change “from” to “of”

Thank you! Done. (line 43)

Line 47: too many bracket, remove bracket before “e.g.” and at the end of sentence

Please note that the inside brackets would be removed when the journal formats the references to hyperlinks

Line 49: change “complementarity” to “the complementarity effects”

We made the suggested change. (line 46)

Line 58-61: The sentence is difficult to understand because it includes a couple of complex structures, please rewrite or brake it down into few sentences

We have now split this complex sentence. (lines 55-58)

Line 57: The paragraph is too short and can be merged with the paragraph above

It is short but, we believe, thematically distinct from the previous one.

Line 65: Coma is missing before “such as” and before “is) in line 66
Line 70: Comas are missing before and after “in turn”
Line 75: coma is missing before “such as”

We have added the missing commas.

Line 72: Energy fluxes and carbon and nutrient cycles are themselves ecosystem functioning, please rephrase or explain what you mean by ecosystem functioning other than those two.

We have rephrased this sentence to clarify that changes in individual functions within an ecosystem can lead to changes in the overall functioning of the system. (lines 67-70)

Line 74: add “plant related” before “functions”

We have added the suggested phrase. (line 72)

Line 76: give the reference at the end of the statement

Done.

Line 80: replace “as plant and soil history” by “the history effects of plant and soil, ”

We have rephrased this sentence. (line 78)

Line 80-82: complex sentence, simplifying or restructuring it might improve readability

This sentence was missing commas; we believe it is now much easier to read. (lines 79-81)

Line 86-90: very long and too complex sentence,

We have restructured this section and…

Line 93: Not clear what you mean by multitrophic functions. I suggest to introduce it before, when you talk about energetics approach, e.g. at the end of the sentence in line 90.

…we have introduced multitrophic functioning. (lines 88-90)

Line 96: Not clear what is the y-axis in Figure 1 (right panel)

We have amended this by adding an axis label.

Results

Line 107: rephrase "of mesocosm soil fauna food-webs"

We have changed the figure caption.

Figure 2: figure letters overlap with the title of y-axis

We have corrected the figure.

Line 114: add "plant" before "history"

Done. (115)

Lines 120-126, and all below: Maybe you can add the table with all the means and 95% HPD, so that you do not have to state them all individually in the text

Thank you for this suggestion, but we would argue that this is not necessary and would add one more place the reader would have to shift their attention to.

Figure 3: Why there are no letters (post-hoc) at the figure a? If no differences you still can identify it by using the same letters across all groups. Also, it is not clear what community biomass mean (figure c), is it plant biomass or is it soil fauna biomass. Until now it was not mentioned before. Already in the introduction section you have to explain more clearly what are the variables for the multitrophic functioning. Add legend to the figure.

Thank you! We have now followed the suggestion to add the letters in panel a. We have also clarified in the figure caption that community biomass refers to the soil fauna community (line 131) but kept the axis label short for space. Finally, we have added a legend to the figure.

Line 144: Which specific Supplement?

We previously forgot to add here the specific section once the supplement was finalized. This also led to that section of the supplement seemingly coming out of the blue as you point out in your last point. See also our response to that point for more details.

Figure 4: Add legend to the figure

We have moved this figure to the supplement, following the suggestion of reviewer 1. We have amended the caption to explain the meaning of the different symbols.

Line 151: “These findings imply that assembly had been leading to”.. Here, please be more specific what assembly processes, e.g. under the soil history effects ? or animal community assembly?

We have removed this sentence, per the reviewer’s comment for lines 151-155

Line 154: add literature reference to the statement “due to a higher 153 metabolic rate of smaller organisms”

Reference added. (line 148)

Lines 151-155: these sentences belong to discussion section rather than to results.

We have removed the interpretive sentence.

Discussion

Line 186-187: it is not clear what authors mean by the “differences in plant density and community composition”. Difference from what or in what? Across treatments? Or in effects of plant diversity vs plant community composition?

We have amended this passage to clarify that we refer to differences of JenaTron with previous experiments. (line 180-182)

Line 194: replace “for” by “to”

Done. (line 188)

Line 226: This statement is repeated in Result section (lines 153-154),

This is no longer the case, after following the reviewer's last “Results” comment.

Lines 241-242: replace “γ and β animal as well as plant 241 diversity” by “γ and β diversity of both animal and plant communities”

We have made the suggested change. (lines 237-238)

Line 249: replace “animal community assembly time” by “the assembly time of animal community”

We have made the suggested change. (lines 245)

Line 253: add literature reference supporting this statement “Given that local diversity is not necessarily declining despite global biodiversity loss”

Reference added. (line 249)

Lines 239-258: The concluding statement almost totally is dedicated to only the lack of biodiversity effects. Authors should also focus here on the effects of soil history, which were the only effects that they found.

We have rephrased the concluding paragraph to emphasize the importance of soil history.

Materials and Methods

Line 331: which specific Supplement are you referring to?

This refers to Supplementary Note 1, which we now mention specifically. (line 335)

Line 362: There is no reference 82 in the reference list

We have added the missing reference.

Statistical analysis: it is not stated why the CWM and community biomass data were log transformed (as shown on Figure 3)

We have added the explanation for this in lines 360-361 of the Methods section.

Fig. S1 in the supplementary file states that authors had two different calculations of energy fluxes, but I could not find the description of this in the method part. It would be great to show such uncertainty analysis, which would show if the results change if different approach is taken for the parameterization of food webs, for example not based on the average body-mass in mesocosm, but randomly sampled from the range of body-mass values in that mesocosm.

(See also our response to point re Line 144) First, we have now removed the confusion regarding this section of the supplement by referencing it explicitly in line 146 of the Results and lines 368-374 of the Methods section *Sensitivity analysis.* We clarify both in the Methods section and in the Table S2 caption that the purpose of this reanalysis is to determine to what extent the observed differences between treatments can be attributed to within-taxon shifts in body-mass or community-level changes that are based on changes of abundance of different taxa. Please also note that the sensitivity analysis now detailed in Supplementary Note 2, while not perturbing body-masses per se, is analogous and broader than what is proposed here.

Reviewer #3 (Remarks to the Author):

This is an interesting study and I am quite intrigued by the incubation of full soil monoliths, and the estimation of soil fauna energy fluxes and ecological function at the community scale. I also agree that the question being addressed is exciting and important. My biggest concern with the study though relates to the concept of a "soil history" when it comes to interpreting the fauna data. For instance, at lines 158-160 the authors state that fauna-driven functioning is promoted in soils with a shared history with plant communities vs soil communities from bare ground (i.e. “no shared history”). I agree that there were clear and interesting differences observed in fauna functioning in soils collected from these two sites (TBE site vs bare soil site), but I do not believe the data support the idea that these differences result from the sites having vs not having a shared plant history. First, it seems very likely that the differences observed in soil fauna function were driven simply by the inherent differences in soil properties between the Trait-Based Experiment site and the bare ground site combined with any management practices used to generate and maintain the desired plant conditions (spp diversity combinations vs bare ground) at those sites. Second, it is stated in the discussion that the bare soil plots did in fact have plants that invaded the plots from field borders. So, in addition to being driven by inherent differences among these two soils, fauna function may also have been shaped by legacy differences in the plant inputs between the TBE and bare soil sites. In other words, given that the plant history treatments had little-to-no impact on fauna functon (neither within or across soil types), the authors should interpret their results less through the lens of “shared history” and should entertain other potential explanations for the differences in function between the TBE and bare soils. True, this is a bit messier than the concise soil-by-plant history framework, but I feel it is much more accurate to the data.

> This point includes concerns that the soil treatments (here mainly TBE with plants vs. bare-ground sites) also come along with differences in (1) abiotic soil properties, and (2) management practices used to generate and maintain the desired plant condition. We now address these concerns by providing some crucial missing details on the maintenance of the field plots and clarifying our interpretation of our findings. We are grateful to the reviewer for highlighting these omissions. First, as we now make clear in lines 276-277, TBE plots and bare ground plots are in the same field, under the same abiotic conditions and soil properties. Second, their plant conditions (specific species composition or bare ground) are similarly maintained by regular weeding of the non-target species (or all species in the case of bare ground) (lines 269-270 and 274-275). Finally, our interpretation of the results (lines 216-221, 198-199 and 206-209) broadly agrees with the reviewer’s emphasis on legacy effects of plant inputs, which, combined with the more stable conditions under the fixed composition of non-bare ground plots, are responsible for biotic and abiotic soil differences. In the revised version of the manuscript, we thus made sure to clearly refer to community-specific histories of soils and plants that originated from the same field site.

I am also not that surprised by the lack of plant history treatment effects given the plant treatments were only in place for 4-5 months although I see this is acknowledged by the authors in the discussion.

> We have been cautiously discussing this finding because multiple mechanisms including the short time interval of the experiment could have been responsible for the lack of plant history effect on the soil functions. Certainly, this will be an interesting point for future studies.

Additional comments:

Line 123 – reference to Fig 1c-e should be Fig 2c-e, correct?

> Yes! Thank you for spotting this. (line 124)

Line 270 - how were the monoliths collected AND were they the same size as cited above (50cm diam x 80cm deep) for the inner lysimeter?

> We now explain in lines 277-278 that the steel lysimeters themselves were used as corers to extract the monoliths so they correspond in size. This technique allows for the extraction of intact monoliths with minimal side effects on soil density and structure.

Line 272-275 - later it states that seeds from the TBE site (i.e. with a shared history) were planted into the monoliths, but were there already any existing plants when monoliths were collected in spring? Also, did any seedlings emerge during the study that were not planted at the onset of the experiment? If so, were they removed by hand?

> Existing vegetation, as well as 5 cm of topsoil containing the seedbank, were removed before establishing the mesocosm communities. Emerging seedlings were weeded out over the first couple of weeks. Only transplanted target plants with the respective plant history were kept. We have included this information in lines 279-280 and 292-293.

Line 275-277 - How were the plots maintained bare for 20 years? In my experience, this typically requires something dramatic like herbicides but it is not explained in the Jena experiment paper referenced. Whatever the method, could there also be legacy effects of such practices on fauna communities unrelated to the introduced history treatments?

> Plots were maintained bare through regular weeding, similar to the non-bare plots. We added this information in lines 269-270 and 274-275.

Line 286-288 - How was this maintained for the 5 month duration? Were unwanted plants removed by hand?

> Yes, this information is now in lines 292-293. Unwanted plants were weeded out.

Results: While I acknowledge there isn't room for this in the main text, I think it would be a great addition to the appendix to include a table of mean abundances for the fauna…some degree of taxon lumping would be fine given the number of taxa observed.

> We have followed this suggestion and added Table S3 in the supplement. Detailed taxa abundances, body-masses and trait information for the taxa that comprise the food-webs are available in the data that are part of the supplementary material.

REVIEWERS' COMMENTS

Reviewer #3 (Remarks to the Author):

I appreciate the author's attention to comments on the first draft of this manuscript. They have taken the time to address my concerns thoroughly, including those related to concerns about level of detail in their methods section that led me to question their ability to separate plant and soil effects as well as any unintended effects stemming from field maintenance and the removal of weeds during the monolith study. I also appreciate their addition of the new fauna table in the appendix. I stand by my original assessment that the full soil monolith and energy flux modeling conducted here are very interesting and that they will be of significance to the field of soil ecology. I feel the manuscript is now suitable for publication in Nature Communications.

> We are happy to hear that the revised version of the manuscript successfully resolves the reviewer's concerns. Once again, we are grateful for their help in identifying points that had required clarification and their generally constructive suggestions.

Reviewer #4 (Remarks to the Author):

This manuscript takes advantage of the long-term JENA experiment, enabling the authors to cleverly separate the impacts of plant community history and soil history on ecosystem functioning mediated by soil fauna. The sampling methodology and the description of the energetic food web modeling are clearly articulated and executed well, demonstrating a significant amount of work, especially given the experiment's relatively short duration of just four months. The inclusion of a sensitivity analysis strengthens the overall analysis, supporting the main conclusion that soil community history enhances multitrophic functioning. The manuscript is well-written, with clear illustrations that enhance readability.

Overall, the authors have done an admirable job addressing the concerns raised by the initial reviewers, and their efforts should be recognized. This work will contribute meaningfully to our understanding of how soil fauna influence ecosystem functioning.

> We greatly appreciate this very positive feedback on our work and are happy to see that this reviewer also agrees that our revision was successful in resolving previous issues.

The one thing that I think could be addressed more effectively is how to introduce the term 'multitrophic functioning.' I'm okay with it being used in the title, but I agree with Reviewer 1 that this term is opaque without context, and the manuscript presents it as if it is a widely accepted term when only a handful of articles have ever used it. However, I do like the idea of using a term that succinctly captures the link between food webs and ecosystem functioning, so one suggestion would be to explicitly define this term early in the Introduction instead of assuming the reader would immediately know what it describes.

Thank you! This is an excellent suggestion. We have now revised the introduction which now includes the following paragraph (lines 72-80):

"In contrast to well-documented temporal changes in the effects of plant diversity on plant-related functions, such as primary productivity or soil microbial activity, it is less well understood how the relationship between plant diversity and functions of the soil fauna community develops over time (15, 21, 22). Soil fauna, with their diverse diets, span several trophic levels (25). Their trophic activity includes herbivory, detritivory, microbivory and predation. Together, these trophic functions regulate processes such as nutrient release from detritus and plant growth (27,31). The multitrophic functioning of the soil community is therefore an integral aspect of overall ecosystem functioning and essential in our understanding of how the latter develops over time (15, 21)."

"
